# Using an e-Delphi consensus technique to develop the Stressful Adverse Veterinary Events Support (SAVES) Framework

**DOI:** 10.1371/journal.pone.0326222

**Published:** 2025-06-24

**Authors:** Julie Gibson, Kate White, Marnie L. Brennan

**Affiliations:** 1 School of Veterinary Medicine and Science, University of Nottingham, Nottingham, United Kingdom; 2 Centre for Evidence-based Veterinary Medicine, University of Nottingham, Nottingham, United Kingdom; Government Medical College and Hospital, INDIA

## Abstract

Co-ordinated approaches to supporting veterinary practitioners in relation to adverse events are needed to mitigate associated practitioner stress and to prevent future occurrence. The overarching aim of this study was to develop a framework that could be used profession wide within the veterinary sector to support practitioners in relation adverse events (the Stressful Adverse Veterinary Events Support Framework; SAVES Framework). A three-round e-Delphi consensus approach was taken. The study used an *a priori* determined panel of 50 stakeholders (50% veterinary practitioners – veterinary surgeons and veterinary nurses registered in the United Kingdom, 28% individuals employed within UK organisations supporting veterinary practitioners and 22% other veterinary care stakeholders). An *a priori* determined consensus agreement level of ≥75% was set. Forty-six recommendations entering the first round were formulated by reviewing two sources (i) The human and veterinary healthcare literature pertaining to the provision of support for practitioners who are involved in adverse events, and (ii) Transcripts from a study by the authors that explored veterinary practitioners’ experiences of and responses to adverse events. Both sources were used to identify recommendations that may be useful to veterinary practitioners and/or highlighted gaps in the current provision of such support. Recommendations were refined by the primary researcher, through discussion with the core research group and with a wider group of stakeholders via a pretesting and pilot phase before being shared with e-Delphi panellists. Panellists were asked to indicate the degree to which they agreed with each recommendation (Likert Scale; 1 = Very strongly disagree, 7 = Very strongly agree) and were informed that indications of 6 or 7 would be considered agreement and indications of 1 or 2 would be considered disagreement. In rounds one and two, panel members were asked to provide feedback regarding the recommendations, which was (i) Used to modify the recommendations or to formulate additional recommendations, and (ii) Qualitatively analysed to generate themes of factors that may influence implementation of support for veterinary practitioners in relation to adverse events. Twenty-nine of the 46 initial recommendations (63%) reached consensus. Feasibility, a multifaceted and flexible approach and the influence of human thought and behaviour were identified as factors participants thought would influence the implementation of such support. The recommendations provide the first evidence-based guidance for specifically supporting veterinary practitioners in relation to adverse events; future work should focus on assessing their implementation and impact.

## Introduction

Stress is defined by changes to individuals’ personal state in response to situations or ‘stressors’ that pose challenge [1]. A foundational conceptualisation of the physiological stress response outlined alarm, resistance and exhaustion stages [2]. Later seminal stress psychology works introduced the idea of stress as a cognitive process and suggested that individuals experiencing stress appraise whether an event is a threat and whether they have the resources to cope before engaging in stress mitigating thoughts or behaviours [3]. The notion of emotional coping was further expanded when research showed that stress can have psychologically positive, as well as detrimental, effects [4]. Therefore, all stressors have the potential to trigger emotional and physiological responses which lead to altered actions and behaviours, but the way stress is experienced and exhibited is dependent on the individual’s evaluation of the inciting cause and the adaptive responses they employ as a consequence. Although stress is most often associated with unpleasant reactions and outcomes within individuals, eustress, from the Greek word ‘Eu’ meaning good, stimulates productive learning and self-growth [5]. Importantly, an individual’s capacity to adapt in a positive way to stressors is influenced not only by personal characteristics but by their interaction with external demands and resources within the environment that they operate [6]. While organisations are legally obligated to address workplace stress [7], the *norm of reciprocity* [8] suggests that employee’s contentedness, commitment and performance are enhanced through provision of support that is perceived to surpasses minimal requirements [9,10].

Adverse events can trigger a specific and defined emotional and behavioural response amongst human healthcare practitioners who are involved [11–13] and are thus a recognised source of occupational stress within the medical profession. Adverse events define instances where unnecessary harm is experienced by patients secondary to medical and surgical complications, errors and mistakes [14]. Such events compromise the safety of patients and have ethical, legal and reputational ramifications. Most poignantly, they raise questions of accountability. Practitioners may, unfoundedly or unfairly, shoulder a burden of responsibility for preventing and for responding constructively in the aftermath of adverse events. This has long been recognised and reasons for it are likely to be complex [15]. It is postulated that emotional impacts occur because of inbuilt traits of those attracted to caring professions. On one hand this can be thought of as an important driver for practitioners to ensure and better their own professional competence. On the other it can lead to unrealistic professional self-expectation, which can be personally damning [15]. However, the role that external factors play cannot be underestimated. Patient dissatisfaction and resulting complaints, investigation processes, organisation- and peer- level scapegoating, may compound the impact of adverse events. This is clearly documented within the human healthcare sphere, where cultures of ‘blame and shame’ prevail and literature suggests that those facing disciplinary action have a significant risk of suffering from depression, anxiety and suicidal ideation [16,17]

For two and a half decades, healthcare practitioners suffering in the wake of adverse events have been referred to as ‘*second victims’* [18]. Provision of appropriate support may mitigate detrimental emotional and behavioural sequalae and the development of longer term second victimhood [19]. Support may also catalyse practitioner and system level growth and positive change as it facilitates learning from such events [20]. Essentially, improvements that are made as a result have the potential to prevent similar adverse event occurrences and emotional sequalae in the future. In the United Kingdom (UK), the fundamentality of such support to patient safety is spotlighted within the National Health Service’s (NHS) Healthcare Safety Investigations’ reports [21] and was recently enveloped within the Patient Safety Incidence Response Framework (PSIRF) [22].

Adverse events [23,24], related ethical challenges [25] and client complaints [26], are also reported to induce an emotional and behavioural stress response amongst veterinary practitioners. The adaptive response appears to broadly mirror that reported amongst their human healthcare counterparts [11–13,27–31]. Support is, likewise, postulated as a factor influencing veterinary practitioners projected emotional and behavioural response [32,33]. In a previous study by the authors, support in the guise of peer mediated reflection was concluded to be a component of veterinary practitioners’ recovery following adverse events. Approaches that facilitate reflective discussion were suggested to aid a culture of learning and improvement, rather than defensiveness amongst practitioners [34]. As understandings of what is needed to optimise patient safety mature, there are calls to develop veterinary specific support strategies that mirror those adopted in human healthcare [35]. Frameworks consist of principles, processes and/ or guidelines that provide expertise that align with the current or future needs of organisations. Development of a framework for the provision of support that aims to mitigate the detrimental impacts of adverse events amongst veterinary practitioners, and harness opportunities for future prevention, would address a current evidence gap.

The overarching aim of this study was to develop such a framework for profession wide use in the veterinary sector (the Stressful Adverse Veterinary Events Support Framework; SAVES Framework). To achieve this aim there were three research objectives. The first was to develop a list of recommendations that could be used nationally in the United Kingdom to guide best practice for the provision of support for veterinary practitioners in relation to their experiences of adverse events. A second was to identify those recommendations deemed most important to the veterinary sector. A consensus building exercise was deemed appropriate given the dearth of evidence surrounding guidance for specifically supporting veterinary practitioners in relation to their involvement in adverse events. A third was to identify any factors that could influence implementation of such support.

## Materials and methods

### Overview of the e-Delphi process utilised in this study

An adaptation of a conventional Delphi consensus building process was utilised in this study. This was deemed appropriate to meet the research aim as Delphi processes facilitate assimilation of the opinions and expertise of relevant stakeholders in a structured and anonymous or ‘quasi-anonymous’ way. The latter means that the identity of those participating remains undisclosed or that their identify is known to other participants and to the researcher(s) but participants do not know how other individual participants responded [36]. Delphi processes are also characterised by an iterative nature where multiple rounds of voting and feedback are employed in an attempt to elicit consensus or agreement amongst a panel of people [37].

‘e-Delphi’ is a term that has been used to describe processes that have been adapted from more conventionally described Delphi methods and employ electronic platforms [38–40]. e-Delphi approaches facilitate diversity and compliance of contributors; by exploiting email and online survey techniques, panellists can remain anonymous and can contribute from any geographical location at a time that is convenient to them [36–38].

The e-Delphi process employed in this study was steered by a core group of researchers. JG, a female veterinary surgeon with nearly 20 years of experience in clinical practice and a PhD student at the School of Veterinary Medicine and Science at the University of Nottingham, led the group. MB, a female veterinary surgeon and veterinary epidemiology specialist educator and researcher and KW, a female veterinary surgeon and educator specialising in analgesia and anaesthesia research and practice, contributed to the e-Delphi process study design and assimilation of findings.

Guidance for researching using Delphi processes within healthcare was used in this study [41,42] and the Accurate Consensus Reporting Document (ACCORD) was used as a reporting checklist for the creation of the manuscript [43]. The research process followed is described in detail below but broadly encompassed: Developing a list of initial recommendations; Planning and recruiting a panel; Pre-defining a consensus level; Designing and distributing survey ‘rounds’ containing recommendations and; Collecting, analysing and presenting the findings.

### Developing an initial list of recommendations to enter the e-Delphi process

**Fig 1:**
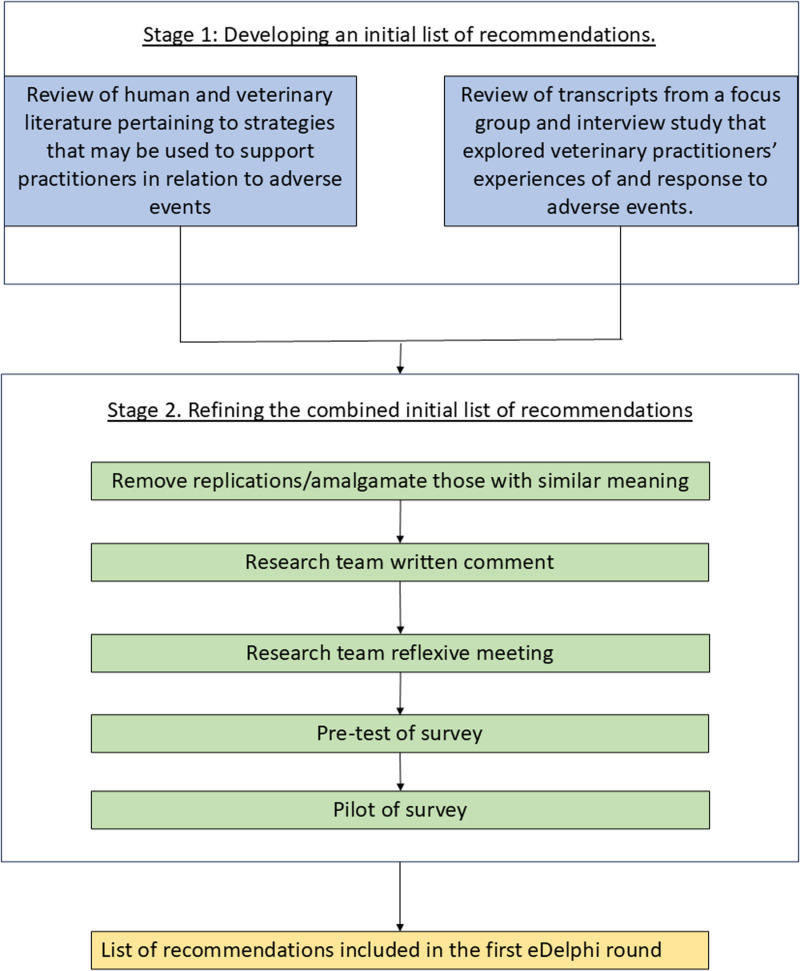
Flow chart demonstrating the process used to develop initial recommendations to enter the first round of the e-Delphi study.

An overview of the process used to develop the recommendations entering the first round of the e-Delphi is shown in Fig 1.

#### Stage 1: Developing an initial list of recommendations.

JG formulated a list of recommendations that drew from review of two sources: (i) Existing human and veterinary healthcare literature pertaining to support for practitioners in relation to stressful workplace and more specifically adverse events. Alongside other literature, the Organisational Staff Support Model (OSSM) (https://secondvictim.co.uk/supporting-second-victims/) was identified as one useful resource from which to draw when developing the recommendations. It was decided to use the structure adopted within the model as a template for the design of the veterinary framework. The model was originally developed by the Yorkshire and Humber Patient Safety Translational Research Centre (YHPSTRC), Yorkshire and Humber Improvement Academy (YHIA) and members of the YHIA’s ‘Just Culture Network’ in the UK. The model suggests a three-pronged approach to supporting human healthcare practitioners in relation to the stress they may experience in relation to events that threaten the safety of patients. It is based on Cooper and Cartwright’s Intervention Strategy for Workplace Stress, which recognises that primary or preventative, secondary or mitigative and tertiary or remedial support measures are necessary [44] (ii) Transcripts from a broad focus group and interview study, previously conducted by the authors, which explored veterinary practitioners’ experiences of adverse events. The decision to incorporate suggestions made within the transcripts was made, due to the very limited pre-existing veterinary specific literature on the topic and to help ensure that the content and language used within the developed recommendations was relevant and comprehensive for the veterinary sector.

#### Stage 2: Refining the recommendations.

The initial list of recommendations was reviewed by JG who removed any replicated suggestions and refined wording to make it as consistent and as relevant to veterinary practice as possible. For example, where an interview or focus group participant had used the word *‘mistake’* or where *‘patient safety incidents’* or *‘stressful events’* were mentioned in the literature, this was replaced with ‘*adverse event’*. A combined list of recommendations was shared with other members of the research team (KW, MB). Each team member independently reviewed the list and provided written feedback to JG who suggested relevant amendments. During a team meeting (JG, MB, KW), the recommendations and amendments were discussed further. Minor changes to wording and the order in which the recommendations would be presented to panellists were agreed. The wording of the recommendations was also reviewed at a pre-testing and piloting stage (see below).

### The e-Delphi panel

#### Identifying and recruiting e-Delphi panellists.

During an initial face-to-face study planning session on 28^th^ June 2022, the steering committee (JG, MB, KW) identified stakeholders relevant to the recommendations being developed. Target stakeholder groups identified as essential for inclusion were (i) veterinary practitioners registered in the UK (veterinary surgeons and veterinary nurses) (ii) UK organisations aiming to provide emotional and/or technical support to veterinary practitioners and (iii) other stakeholders relevant to veterinary patient care. Defined eligibility for inclusion within groups and purposive and snowballing sampling techniques employed to recruit panellists were as follows:

***Veterinary practitioners:*** Veterinary surgeons or registered veterinary nurses currently working in a clinical role in the United Kingdom, including those in leadership and management positions, were eligible for inclusion in the veterinary practitioner group. Individuals who had given consent to be contacted about further research following participation in a prior qualitative study, which explored veterinary practitioners’ experiences of adverse events and informed development of the initial recommendations, were invited via email to become panellists. The email contained information about the purpose of the e-Delphi study, including contact details of the primary researcher (JG). The invitation email explained that a snowballing technique to recruitment was being used and the potential participants were asked to forward the invitation to eligible colleagues who might be interested in participating.

***Veterinary practitioner support organisations:*** Eligibility for inclusion in the veterinary practitioner support stakeholder group, was defined as an individual currently employed or volunteering at an organisation specifically aiming to provide emotional, financial, legal, or regulatory advice and/or resources for veterinary practitioners. Organisations offering a combination of types of support fell within the eligibility criteria. Recruitment of panellists occurred from six support organisations: (i) The British Veterinary Association (BVA) (https://www.bva.co.uk/), the largest membership community for the veterinary profession in the UK, which supports veterinary surgeon members by providing professional advice, training and resources (ii) The British Veterinary Nursing Association (BVNA) (https://bvna.org.uk/), the representative body for veterinary nurses in the UK (iii) The Society for Practising Veterinary Surgeons (SPVS Ltd) (https://spvs.org.uk/), a specialist division of the BVA focused on supporting veterinary practices to be sustainable, profitable, happy and productive places work within, (iv) Vetlife (https://www.vetlife.org.uk/), an independent charity that provides free and confidential advice to anyone in the veterinary community who has emotional, health or financial concerns, (v) ProfCon Investigation Support (https://vetsupport.me/profcon/), a confidential service, which is funded by the regulatory body for the UK veterinary profession called the Royal College of Veterinary Surgeons (RCVS), but is independently run by volunteers who support veterinary practitioners who are going through the RCVS professional conduct investigation process, and (vi) The Veterinary Defence Society (VDS) (https://www.thevds.co.uk/), a mutual insurance company (run by experienced veterinary surgeons on behalf of the UK veterinary profession), which insures veterinary practitioners against the effects of claims of negligence, provides legal representation and supports members through provision of advice, guidance, publications and training.

***Other veterinary care stakeholders:*** It was decided that veterinary clients, educationalists and the RCVS would be included in a final overarching stakeholder group. *Veterinary clients* who had participated in a prior qualitative study that explored clients’ experiences of veterinary adverse events and had given consent to be contacted about further research were invited to become panellists. The email contained information about the purpose of the eDelphi study, including contact details of the primary researcher (JG). Individuals employed as clinical and/or professional skills lecturers within a UK veterinary school and those employed at the RCVS were also eligible for inclusion within this group. Publicly available email addresses of eligible individuals were located online and invitations to participate were sent until the required number of panellists accepted.

### Panel size and composition

Target stakeholder group proportions were decided prior to recruitment to prevent recommendations reaching consensus based on agreement from any single stakeholder group alone. It was deemed essential that veterinary practitioners should represent a majority stakeholder group due to the intended end purpose of the recommendations. Veterinary practitioner support was considered to be the next most important stakeholder group due to a broad understanding of the support needs of practitioners. The purpose was to ensure that a recommendation could only reach consensus for inclusion or exclusion if (i) a proportion of all three stakeholder groups agreed or (ii) the majority of veterinary practitioners and veterinary practice support agreed.

Due to the purposive sampling technique employed, a target panel size of 50 was considered practical based on numbers of veterinary practitioners and clients who had consented to being contacted about further research when participating in prior qualitative studies exploring their experiences of adverse events and accounting for a proportion of expected attrition. The proposed panel composition can be viewed in Table 1.

**Table 1:** Proposed panel composition for an eDelphi study that sought to build consensus on a list of recommendations that may be used to support veterinary practitioners in relation to their experiences of adverse veterinary events.

Stakeholder group		Proposed percentage of total panel (%)	Proposed number of panellists
Veterinary practitioners		50	25
Veterinary practitioner support		28	14
Other stakeholders to veterinary care	RCVS	4	2
	Clients	10	5
	Educationalist	8	4
Total		100%	50

### Consensus

A consensus level of equal to or more than seventy five percent was decided *a priori.*

(i) Recommendations reaching the pre-defined consensus level of either agreement or disagreement within any given round would be automatically included or excluded from the SAVES Framework respectively and would not feature in further rounds.
(ii) Recommendations failing to reach this predefined consensus level within any round would be included in the next round in an unchanged or amended format.
(iii) Recommendations failing to reach this consensus level on completion of all rounds would automatically be excluded from the SAVES framework.

The process used to amend recommendations between rounds and the method used to determine the level of agreement and disagreement amongst panellists is detailed in the following ‘Questionnaire rounds’ section of this manuscript.

### Design and distribution of the e-Delphi survey

#### The survey platform.

The Online Surveys platform (https://www.onlinesurveys.ac.uk/) was used to automate distribution of the recommendations and to collate the responses. Only the first author (JG) had access to the Online Survey dashboard and was privy to the identity and email addresses of panellists. The survey platform assigned each panellist a token (letters and numbers), which was captured by a pre-set hidden question designed to allow JG to track responses.

#### Pre-testing and piloting round.

The online version of the survey was circulated to members of the Centre for Evidence-Based Veterinary Medicine (CEVM), a multi-disciplinary research group at the University of Nottingham (https://www.nottingham.ac.uk/cevm/). Eight members responded. This pre-test phase allowed the survey distribution method to be checked, the content screened for errors and a final wording and comprehension check to be performed. Responses were used to rectify issues with online functionality, layout, spelling and grammar comprehensibility of the recommendations. Guidance about publishing accessible documents was suggested by a pre-test respondent and was used to refine the design following this stage (https://www.gov.uk/guidance/publishing-accessible-documents).

A pilot was then conducted to assess face validity of the survey. Three individuals completed the pilot questionnaire. Each individual was representative of at least one of the stakeholder groups; one clinically practicing veterinary surgeon concurrently employed by the Veterinary Defence Society, volunteering at VetLife and the British Veterinary Association; one veterinary educationalist employed at a Veterinary School in the United Kingdom; and one veterinary client. No changes were deemed necessary following the pilot stage.

### Ethical considerations

Ethics approval for this study was granted by the University of Nottingham, School of Veterinary Medicine and Science ethics committee, approval numbers 2444 180724, 3184 200528, 3790230215, 3858 230524. Although the panel was planned in advance, recruitment of the participants began when the first survey was distributed (20^th^ June 2023). Information regarding the purpose of the study, research ethics, confidentiality, expected time it would take to compete the task, the closing date and researcher contact details were provided at the start of each questionnaire. Panellists had to provide informed consent to participate in order to electronically enter each questionnaire after being briefed that post participation withdrawal from the study was not possible due to anonymity. No additional panellists were recruited following the first questionnaire. Panellists completed each questionnaire anonymously and were not informed of the identity of any other panellist at any stage before, during or after the process.

### Questionnaire rounds

#### Round 1.

The first questionnaire was distributed on 20^th^ June 2023 and was initially open for a period of fourteen days. Personalised email reminders were sent to non-responding panellists on days five and ten. A final personalised reminder was sent on day thirteen, when panellists were given the opportunity to request an extension to allow them to complete the questionnaire. To allow for collation of results and design of the next questionnaire, a period of two weeks was allowed to lapse before launching the next round questionnaire.

Panellists were presented with the forty-six initial recommendations and instructed to indicate to what extent they agreed with recommendations aimed at supporting veterinary practitioners in relation to their involvement in adverse events in practice using a Likert scale (1–7; 1 indicating very strong disagreement, 7 indicating very strong agreement). Panellists were also asked to suggest amendments to the existing recommendations, suggest new recommendations and to make general comments about the recommendations and research process in free text response boxes as appropriate. Consensus levels and potential outcomes were described to the panellists as follows:

*“If equal to or more than seventy-five percent of panellists indicate 6 or 7, the recommendation will reach consensus for agreement and the recommendation will be automatically included in the SAVES framework and not be included in further rounds.*

*If equal to or more than seventy-five percent of panellists indicate 1 or 2, the recommendation will reach consensus for disagreement and the recommendation will be automatically excluded from the SAVES framework and will not be included in further rounds.*

Likert scores (and calculated percentages) for each recommendation were transferred to a word document, where they were tabulated along with confirmation of whether the recommendation had reached consensus for inclusion or exclusion and associated free text comments. Identifying data was removed from the free text data. Free text comments were reviewed by JG. Modifications to initial recommendation(s) not reaching consensus were made if more than one panellist made the same or similar suggested amendment. Additional recommendations were formulated if more than one panellist made the same or similar suggestion. Results were reported to the steering committee (MB, KW) who reviewed the recommendation list prior to the initiation of the second e-Delphi round.

#### Round 2.

The second round was distributed on 23^rd^ July 2023 and was initially open for a period of four weeks, with personalised email reminders being sent to non-respondent panellists on days ten, twenty-one and twenty-eight. A link to a pdf. word document listing recommendations that had reached consensus in the previous round was provided for reference, but panellists could not comment on or re-rate these recommendations. Panellists were presented with the recommendations failing to reach consensus in an unchanged or amended format as appropriate. Panellists were again asked to indicate their level of agreement with each recommendation (1–7; 1 indicating very strong disagreement, 7 indicating very strong agreement). Opportunity to comment within free text response boxes was again provided. The method of collating the findings to produce a list of recommendations to be entered in the next round was the same used as in round 1.

#### Round 3.

The third questionnaire was distributed on 26^th^ September 2023 and was open for five weeks with personalised email reminders being sent to non-respondent panellists on days seven, fourteen, twenty-one and twenty-eight. Panellists were again provided with a link to a pdf that contained the list of recommendations that had reached consensus in round 1 and 2. In addition, the percentage of panellists indicating 6 or 7 on the Likert scale for the recommendation was shown against each. Recommendations not reaching consensus in round 2 entered into round 3 in an unchanged format but the percentage of panellists indicating 6 or 7 on the Likert scale was provided against each. Panellists were asked to re-consider each recommendation in light of the percentage of panellists indicating 6 or 7 on the Likert scale in round 2. Panellists were not given opportunity to add free text comments about the recommendations in round 3. The method of collating the findings to produce a list of recommendations reaching consensus and a list failing to reach consensus at the end of round three was the same as in round 1 and 2.

#### Further analysis of the free text comments.

At the end of the three rounds, free text comments from round one and two, were screened and identifying information was redacted prior to uploading to Microsoft Excel™ [45]. The comments were analysed using a four-stage inductive reflexive thematic analysis approach as described by Braun and Clarke [46,47]. JG read all comments twice in a familiarisation stage (stage one), then generated and assigned codes reflecting the semantic meaning of the text (stage 2). Codes with shared meaning were grouped together into themes (stage 3), which were then discussed and reviewed by all authors to enhance rigour of the interpretative process (stage 4).

## Results

### Development of the initial recommendations

Forty-six recommendations were formulated and presented to panellists in the first round. A complete list of the recommendations and the source(s) from which they were formulated is presented in Table 2.

**Table 2:** Initial list of a total of 46 recommendations, comprised of suggestions for Primary (n = 26; green cells) Secondary (n = 14; yellow cells) and Tertiary (n = 6; blue cells) support, entering round 1 of the e-Delphi process which aimed to reach consensus on a list of recommendations that could be used to support veterinary practitioners in relation to adverse events.

Recommendations entering the first round of the eDelphi process	Evidence source(s)	
	Existing human and veterinary literature pertaining to strategies that may be used to support practitioners in relation to adverse events.	Transcripts from a study that explored veterinary practitioners’ experiences of and response to adverse events.
1. Veterinary practitioners should receive basic education/training in the causes and types of adverse event.		✔
2. Veterinary practitioners should receive basic education/training in adverse event review techniques.		✔
3. Veterinary practitioners should receive training in communicating with clients in the aftermath of an adverse event.	✔	✔
4. Veterinary practitioners should receive education about veterinary regulation, governance and legal responsibilities in relation to adverse events.		✔
5. Veterinary practitioners should receive education/training about the emotional and professional impacts of adverse events.	✔	✔
6. Veterinary practitioners should receive regular resilience training.	✔	
7. Regular veterinary practitioner group meetings, where clinical aspects of cases are discussed, should be conducted within practice.	✔	✔
8. Regular veterinary practitioner group meetings, where the social, emotional and professional aspects of clinical work are discussed, should be conducted within practice.	✔	✔
9. Regular veterinary practitioner group meetings, where ethical aspects of clinical work are discussed, should be conducted within practice.	✔	✔
10. Veterinary practitioner group meetings should be compulsory to attend, or provision made for those unable to attend to be updated on the meeting discussions.		✔
11. Veterinary practitioner group meetings should always be led by facilitators who are trained in conducting the specific meeting type.		✔
12. Veterinary practitioner group meetings should always be prescheduled.		✔
13. Veterinary practitioner group meetings should always be conducted within working hours.		✔
14. Pre-scheduled meetings between one practitioner and one equally or more experienced practitioner, where any aspect of clinical work can be discussed, should be conducted within the practice.	✔	✔
15. Veterinary practitioners should have the ability to discuss any social, emotional, professional, ethical or clinical aspect of a case with an equally or more experienced practitioner on a one-to-one basis at any time.		✔
16. Written comments/suggestions/concerns from veterinary practitioners about any social, emotional, professional, ethical or clinical aspects of cases should be encouraged through the provision of physical or virtual comment boxes within the practice.		✔
17. Veterinary practitioners contributing written comments/suggestions/concerns regarding social, emotional, professional, ethical or clinical aspects of care via comment boxes should have the ability to remain anonymous.		✔
18. Approaches used for reviewing and learning from adverse events should be pre-agreed by veterinary practitioners.		✔
19. Designated roles and responsibilities for recording and reviewing adverse events should be pre-agreed by veterinary practitioners within a practice.		✔
20. Approaches to responding to adverse events should be reviewed at predetermined time periods within a practice (e.g., quarterly, bi-annually, annually).		✔
21. Acceptable and unacceptable veterinary practitioner behaviours surrounding adverse events and adverse event review should be pre-agreed by veterinary practitioners working within a practice.		✔
22. Adverse event review processes, roles, responsibilities and expected conduct should be clearly documented and accessible for reference by veterinary practitioners.	✔	✔
23. Veterinary practitioners’ experiences should be used as feedback to regularly review processes, roles, responsibilities and conduct in relation to adverse event review.	✔	✔
24. Veterinary practitioners should pre-agree designated role responsibilities for communicating with owners of animals affected by an adverse event within a practice.		✔
25. A written policy or ‘Charter’ explaining the rights and responsibilities of both veterinary practitioners and veterinary clients in relation to adverse events should be clearly displayed within veterinary practices.		✔
26. Information regarding professional and emotional support available from external bodies (e.g., professional body advice lines, BVA, VDS, Vetlife) should be clearly visible within veterinary practitioners’ workplace environments.	✔	✔
27. Immediately following an adverse event, a one-to-one discussion between an involved veterinary practitioner(s) and a peer trained in Psychological First Aid (PFA) should take place to ensure the practitioner(s) is psychologically safe.	✔	
28. One-to-three weeks after an adverse event, a group discussion led by a peer trained in Critical Stress Incident Debriefing, comprising veterinary practitioners involved and those with knowledge of the event, should take place to facilitate reflection on both the emotional and clinical aspects of the event.	✔	
29. One week to several months after an adverse event, a group discussion led by a peer trained in Clinical Ethics Debriefing (CED), comprising veterinary practitioners involved and those with knowledge of the event, should take place to facilitate reflection on ethical aspects of the event.	✔	
30. At a mutually agreed time after the event, a one-to-one discussion between an individual involved in an adverse event and an experienced clinician should take place as part of the individual’s personal and professional learning and development.	✔	
31. Adverse events should be reviewed during prescheduled meetings which are conducted during working hours.		✔
32. Adverse events should be reviewed using a standardised template (e.g., those used in root cause analysis/fishbone diagram/Five Why’s/Six Sigma etc.).		✔
33. Adverse event review should be conducted within a meeting which is open to all veterinary practitioners regardless of their degree of involvement in or knowledge of the event.		✔
34. Adverse event review meetings should be led by a trained facilitator.		✔
35. Adverse event review findings, learning and action points should be recorded and stored in a form accessible for reference by veterinary practitioners within a practice.		✔
36. Adverse event review findings should not be considered during performance review or practice disciplinary proceedings.		✔
37. Veterinary practitioners who are involved in an adverse event should always be provided with clear information regarding review and/or investigation processes.	✔	
38. Veterinary practitioners who are involved in an adverse event should always be provided with clear information about the potential emotional and professional impact.	✔	
39. Veterinary practitioners involved in adverse events should always be signposted to specific emotional support providers (e.g., Vetlife, VetSupport).	✔	✔
40. Veterinary practitioners involved in adverse events should always be signposted to professional body advice lines (e.g., VDS, BVA etc).	✔	✔
41. Veterinary practices should facilitate reasonable adjustments to duties that are requested by practitioners who are impacted emotionally and/or professionally by involvement in an adverse event.	✔	
42. Veterinary practices should facilitate provision of reasonable mentoring or additional training requests by practitioners who are impacted emotionally or professionally by involvement in an adverse event.	✔	
43. Veterinary practices should regularly review the ongoing support needs of practitioners who are emotionally and/or professionally impacted by involvement in an adverse event	✔	
44. Veterinary practices should provide clear information to practitioners who are emotional and/or professionally impacted by involvement in an adverse event, regarding the availability of workplace programs that offer free and confidential assessments, short term counselling, referrals and follow-up (i.e., Employee Assistance Programs).	✔	
45. Veterinary practitioners should be offered opportunity to contribute to practice discussions about their personal experiences of being involved in an adverse event.	✔	✔
46. Veterinary practitioners should be offered opportunity to lead an improvement project based on lessons learnt from being involved in an adverse event.	✔	✔

Primary or preventative recommendations (n=26; green cells)

Secondary or mitigative recommendations (n=14; yellow cells)

Tertiary or remedial recommendations (n=6; blue cells)

### Panellists recruited

Of 50 individuals contacted in line with the proposed panel composition and recruitment criteria described, 48 accepted the invitation to participate in the e-Delphi process. One declined and 1 did not respond. Recruitment of a further 2 eligible panellists by snowball sampling was successful and the proposed panel composition was achieved as shown previously in Table 1. All 50 panellists provided informed consent to participate throughout the entirety of the study.

### Delphi rounds

**Fig 2:**
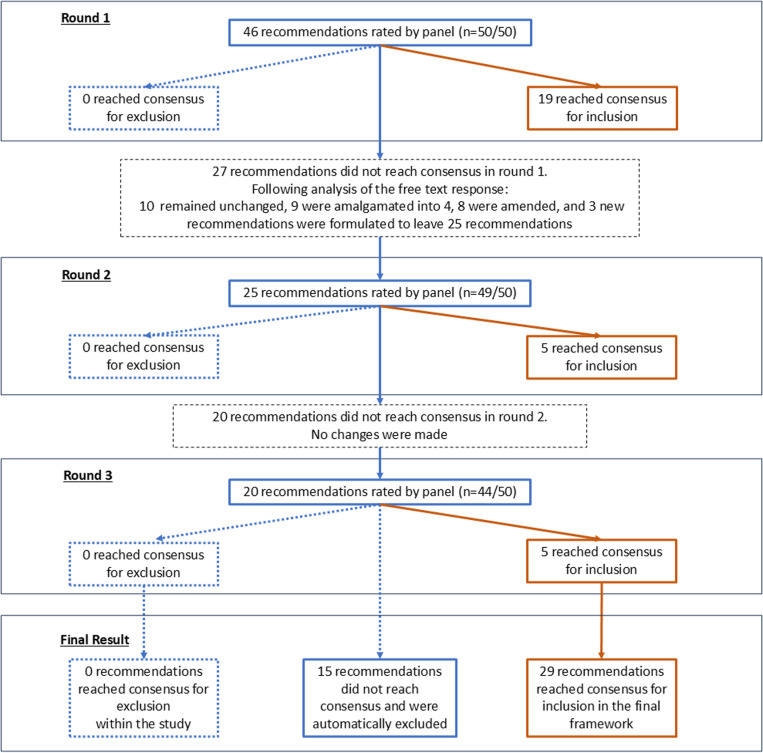
Flow chart outlining the results of the three-round e-Delphi process that was used to develop consensus on recommendations aimed at supporting veterinary practitioners in relation to their experiences of adverse events.

An overview of the results of the three questionnaire rounds conducted as part of the e-Delphi is shown in Fig 2 and further detail can be found at S1 File.

#### Round 1.

Almost all panellists (48/50; 96%) completed the survey in the planned four-week period (20^th^ June-18^th^ July). On request, 2/50 (4%) of the panellists were granted a time extension of a week in order for them to contribute. Overall, 50/50 (100%) of panellists completed all parts of the first-round.

Nineteen of the 46 recommendations reached consensus for inclusion. No recommendations reached consensus for exclusion and 27/46 recommendations did not reach consensus. Based on the free text responses, 10 of these 27 recommendations remained unchanged, 8 recommendations were amended, 9 were amalgamated into 4, and 3 new recommendations were formulated. Details of the amended, amalgamated and new recommendations formulated after round 1 are shown in Table 3.

**Table 3:** *Amended (n = 8), ^amalgamated (n = 9 into 4) and &new (n = 3) recommendations formulated at the end of round 1 along with example free text comments leading to the change.

Recommendations of those that did NOT reach consensus in round 1 (n = 27/46) that were amended (n = 8/27) or amalgamated with other(s) (n = 9/27) prior to entering round 2.	Example free text comment provided by panellists in round 1 that was used to amend or amalgamate recommendations or to develop a new recommendation.	Recommendations to enter round 2 (n = 25)
Veterinary practitioners should receive regular resilience training.	*resilience training needs definition* *I thinking resilience training needs to be specific to an event/moment rather than just a person* *if including resilience training, we could also be providing training on systems and how to improve those, make them more robust, review and update to take some of the emphasis off the individual.*	*Veterinary practitioners should receive training about strategies that veterinary teams may collectively use to withstand or recover from personal and professional impacts of adverse events (e.g., team resilience training).
Regular veterinary practitioner group meetings, where the social, emotional and professional aspects of clinical work are discussed, should be conducted within practice	*I agree these should be discussed but it may be there is an alternative space to discuss them not in the practice which may suit that group, I would sooner see a practice meet out of the practice to discuss than not meet at all.*	^Practitioners should be given the opportunity to attend veterinary practitioner group meetings where reflection and discussion of the non-clinical aspects (social, emotional, professional and ethical) of veterinary care is encouraged
Regular veterinary practitioner group meetings, where ethical aspects of clinical work are discussed, should be conducted within practice		
Veterinary practitioner group meetings should be compulsory to attend, or provision made for those unable to attend to be updated on the meeting discussions	*I do not believe meeting should be compulsory. Doing so may compromise individuals who do not have the capacity at a given time and may impact adversely on their emotional and mental wellbeing*	*Veterinary practitioners should be given the opportunity to attend group meetings, or provision made for them to receive an update on any meetings they do not attend.
Veterinary practitioner group meetings should always be led by facilitators who are trained in conducting the specific meeting type.	*I don’t think you can say always because I’m pretty sure that’s not possible*	*Veterinary practitioner group meetings should be led by facilitators who are trained or have experience in conducting the specific meeting type.
Veterinary practitioner group meetings should always be prescheduled.	*Many of the above recommendations refer to pre-agreed or pre-scheduled systems and processes. Whilst I think it’s really important to have established protocols in place, I also think it’s important to have a degree of flexibility so that a team can react to the situation in front of them*	*Veterinary practitioners should be notified in advance about the intended time, format and purpose of group meetings where clinical and non-clinical aspects of work are to be discussed.
Veterinary practitioner group meetings should always be conducted within working hours.	*Finding time outside of work where there are no other distractions may be beneficial?* *ideal but not essential, could be done outside of work if agreed by both parties*	*Veterinary practitioner group meetings should be offered within working hours but may be conducted out of working hours if veterinary team members are in agreement.
Pre-scheduled meetings between one practitioner and one equally or more experienced practitioner, where any aspect of clinical work can be discussed, should be conducted within the practice.	*In my work we have our team leaders as a kind of buddy and monthly 1:1 meetings where all aspects of life private and work are discussed. We also always have them as point of contact for any issues that we need to report. It means we have a safe place to talk about things by someone who understands and can help*	^One-to-one discussions about any clinical or non-clinical aspects of work (social, emotional, ethical, professional) should be encouraged via the provision of a ‘buddy’ system within practices.
Veterinary practitioners should have the ability to discuss any social, emotional, professional, ethical or clinical aspect of a case with an equally or more experienced practitioner on a one-to-one basis at any time.		
Approaches used for reviewing and learning from adverse events should be pre-agreed by veterinary practitioners.	*The team should be encouraged to develop resources that work best for them and their practice, this may involve drawing on standardised templated listed but it is more important that they work for the practice rather than comply to a previously published format. I worry about being too prescriptive.*	^Adverse event review processes, roles, responsibilities and expected conduct should be pre-agreed by veterinary practitioners working within a practice.
Determining what is acceptable and unacceptable veterinary practitioner behaviours surrounding adverse events and adverse event review should be pre-agreed by veterinary practitioners working within a practice.		
Approaches to responding to adverse events should be reviewed at predetermined time periods within a practice (e.g., quarterly, bi-annually, annually).	*I would add in who should do it must be predetermined otherwise it won’t get done in a busy practice.*	*Adverse event review processes, roles, responsibilities and expected conduct should be reviewed at predetermined time periods within a practice (e.g., quarterly, bi-annually, annually).
	*Perhaps inclusion of training in how to support colleagues going through the aftermath of an adverse event.*	&Veterinary practitioners should receive training in how to support colleagues who may be emotionally and/or professionally affected in the aftermath of an adverse event.
	*Communicating the risk of adverse events to clients is something that we should be more open about as practices. But I feel there is always great pressure to reassure owners that worst case scenario won’t happen. As much as reassurance is important, discussing things that can be exceptions is important and we all tend to do it if adverse events are common but no so much when they are a bit rarer.*	&Veterinary practitioners should receive training in how to communicate the risk of adverse events to clients
Immediately following an adverse event, a one-to-one discussion between an involved veterinary practitioner(s) and a peer trained in Psychological First Aid (PFA) should take place to ensure the practitioner(s) is psychologically safe.	*The demand for all these different meetings within the practice day is becoming unsustainable. We cannot have meetings and do clinical work at the same time. Scheduling time to perform these is difficult and whilst every attempt to have these meetings is made the great practical difficulty is having them when the clinical work does and needs to take precedent. Some could be done at the same time.* *I believe this is important to facilitate honest reflection. It’s important that your thoughts cannot be used as a stick to beat you. Particularly as those thoughts maybe half formed an emotionally weighted*	^Veterinary practitioners who are involved in an adverse event should be offered opportunity to discuss the non-clinical (social, emotional, ethical and professional) aspects of the event within a group meeting at mutually agreed times in the aftermath (group debriefing).
One-to-three weeks after an adverse event, a group discussion led by a peer trained in Critical Stress Incident Debriefing, comprising veterinary practitioners involved and those with knowledge of the event, should take place to facilitate reflection on both the emotional and clinical aspects of the event.		
One week to several months after an adverse event, a group discussion led by a peer trained in Clinical Ethics Debriefing (CED), comprising veterinary practitioners involved and those with knowledge of the event, should take place to facilitate reflection on ethical aspects of the event.		
Adverse event review meetings should be led by a trained facilitator.	*Are we going to have someone with this training on hand??*	*Where possible, adverse event review meetings should be led by a trained facilitator.
Adverse event review findings, learning and action points should be recorded and stored in a form accessible for reference by veterinary practitioners within a practice.	*Anonymity engenders trust and helps the distressed practitioner. It’s a must for this*	*Adverse event review findings, learning and action points should be recorded and stored in an anonymised secure form for reference by veterinary practitioners within a practice.
	*VetSafe covers a lot of this* *I think engagement with centralised adverse event reporting systems should be a mandated part of clinical governance requirements!*	&Veterinary practitioners should be encouraged to enter details of adverse events on a centralised reporting system (such as VetSafe; VDS) and use this to review adverse events quarterly.

Primary or preventative recommendations (green cells)

Secondary or mitigative recommendations (yellow cells)

* Amendment of an initial recommendation as a result of free text responses provided by panellists in round 1 (n = 8/25).

^ Amalgamation of two or more initial recommendations as a result of free text responses provided by panellists in round 1 (n = 4/25).

& New recommendation formulated in response to free text comments following round 1 (n = 3).

(NB. 10 recommendations not listed here proceeded from round 1 to round 2 in an unchanged format. )

#### Round 2.

The majority of panellists (49/50; 98%) completed all parts of round 2 within the five-week open period (23^rd^ July-28^th^ August 2023). The panellist not completing the survey belonged to the veterinary practitioner support organisation group. Five further recommendations reached consensus for inclusion in the second round and no recommendations reached consensus for exclusion. Four of five of the recommendations reaching consensus in round 2 were recommendations that had been amended (2 recommendations), amalgamated (1 recommendation), or newly formulated (1 recommendation) based on free text comments at the end of round 1.

#### Round 3.

Most panellists (n = 44/49; 90%) completed all parts of round 3 within the five-week open period (26^th^ September-31^st^ October 2023). Panellists not completing round 3 were represented across stakeholder groups (veterinary practitioners, n = 2; veterinary practitioner support organisations; n = 2; other veterinary stakeholders, n = 1). The composition of the remaining contributing panel was 52% veterinary practitioners; 25% veterinary practitioner support organisations; 23% other veterinary stakeholders).

Five further recommendations reached consensus for inclusion in the second round and no recommendations reached consensus for exclusion. Again, 4/5 of the recommendations reaching consensus in round 2 were recommendations that had been amended (1 recommendations), amalgamated (1 recommendation), or newly formulated (2 recommendations) based on free text comments at the end of round 1.

Details of the final list of recommendations reaching consensus, along with indication of whether each recommendation is preventative (primary), mitigative (secondary) or remedial (tertiary) are presented in Table 4. For a list of the recommendations that did not reach consensus for inclusion within the final framework please see S2 File.

**Table 4:** Details of 29 recommendations that reached consensus for inclusion within a framework intended to support veterinary practitioners in relation to their experiences of adverse events (SAVES).

Final list of recommendations reaching consensus for inclusion within a framework intended to support veterinary practitioners in relation to their experiences of adverse events (n = 29)	Round reaching consensus	Number (percentage) panellists
Veterinary practitioners should receive basic education/training in the causes and types of adverse event.	1	44/50 (88)
Veterinary practitioners should receive basic education/training in adverse event review techniques.	1	44/50 (88)
Veterinary practitioners should receive training in communicating with clients in the aftermath of an adverse event.	1	44/50 (88)
Veterinary practitioners should receive education about veterinary regulation, governance and legal responsibilities in relation to adverse events.	1	38/50 (76)
Veterinary practitioners should receive education/training about the emotional and professional impacts of adverse events.	1	38/50 (76)
Adverse event review processes, roles, responsibilities and expected conduct should be clearly documented and accessible for reference by veterinary practitioners.	1	38/50 (76)
Veterinary practitioners’ experiences should be used as feedback to regularly review processes, roles, responsibilities and conduct in relation to adverse event review.	1	39/50 (78)
Information regarding professional and emotional support available from external bodies (e.g., professional body advice lines, BVA, VDS, Vetlife) should be clearly visible within veterinary practitioners’ workplace environments.	1	38/50 (76)
Regular veterinary practitioner group meetings, where clinical aspects of cases are discussed, should be conducted within practice.	1	42/50 (84)
*Veterinary practitioners should receive training about strategies that veterinary teams may collectively use to withstand or recover from personal and professional impacts of adverse events (e.g., team resilience training).	3	34/44 (77.3)
*Veterinary practitioner group meetings should be offered within working hours but may be conducted out of working hours if veterinary team members are in agreement.	2	38/48 (77.6)
*Veterinary practitioners should be notified in advance about the intended time, format and purpose of group meetings where clinical and non-clinical aspects of work are to be discussed.	2	38/49 (77.6)
^Practitioners should be given the opportunity to attend veterinary practitioner group meetings where reflection and discussion of the non-clinical aspects (social, emotional, professional and ethical) of veterinary care is encouraged.	3	33/44 (75)
&Veterinary practitioners should receive training in how to support colleagues who may be emotionally and/or professionally affected in the aftermath of an adverse event.	3	33/44 (75)
&Veterinary practitioners should receive training in how to communicate the risk of adverse events to clients	2	38/49 (77.6)
At a mutually agreed time after the event, a one-to-one discussion between an individual involved in an adverse event and an experienced clinician should take place as part of the individual’s personal and professional learning and development.	1	40/50 (80)
Adverse events should be reviewed during prescheduled meetings which are conducted during working hours.	3	37/44 (84)
Veterinary practitioners who are involved in an adverse event should always be provided with clear information regarding review and/or investigation processes.	1	43/50 (86)
Veterinary practitioners who are involved in an adverse event should always be provided with clear information about the potential emotional and professional impact.	1	38/50 (76)
Veterinary practitioners involved in adverse events should always be signposted to specific emotional support providers (e.g., Vetlife, VetSupport).	1	40/50 (80)
Veterinary practitioners involved in adverse events should always be signposted to professional body advice lines (e.g., VDS, BVA etc).	1	38/50 (76)
*Adverse event review findings, learning and action points should be recorded and stored in an anonymised secure form for reference by veterinary practitioners within a practice.	2	39/49 (79.6)
^Veterinary practitioners who are involved in an adverse event should be offered opportunity to discuss the non-clinical (social, emotional, ethical and professional) aspects of the event within a group meeting at mutually agreed times in the aftermath (group debriefing).	2	41/49 (83.7)
&Veterinary practitioners should be encouraged to enter details of adverse events on a centralised reporting system (such as VetSafe; VDS) and use this to review adverse events quarterly.	3	35/44 (79.5)
Veterinary practices should facilitate provision of reasonable mentoring or additional training requests by practitioners who are impacted emotionally or professionally by involvement in an adverse event.	1	41/50 (82)
Veterinary practices should regularly review the ongoing support needs of practitioners who are emotionally and/or professionally impacted by involvement in an adverse event.	1	41/50 (82)
Veterinary practices should provide clear information to practitioners who are emotional and/or professionally impacted by involvement in an adverse event, regarding the availability of workplace programs that offer free and confidential assessments, short term counselling, referrals and follow-up (i.e., Employee Assistance Programs).	1	38/50 (76)
Veterinary practitioners should be offered opportunity to contribute to practice discussions about their personal experiences of being involved in an adverse event.	1	44/50 (88)
Veterinary practitioners should be offered opportunity to lead an improvement project based on lessons learnt from being involved in an adverse event.	1	39/50 (78)

Primary or preventative recommendations (n=15; green cells)

Secondary or mitigative recommendations (n=9; yellow cells)

Tertiary or remedial recommendations (n=5; blue cells)

Initial recommendations reaching consensus unchanged (n = 20)*Recommendation resulting from amendments to an initial recommendation as a result of free text responses provided by panellists in round 1 (n = 4)

^ Recommendation resulting from amalgamation of initial recommendations as a result of free text responses provided by panellists in round 1 (n=2)

& New recommendation formulated in response to free text comments following round 1 (n=3)

#### Further analysis of the free text comments.

Throughout the entirety of the e-Delphi process, panellists contributed a total of 226 free text comments comprising 8468 words (Round 1 = 151 comments, 5506 words; Round 2 = 75 comments, 2860 words). Three themes relating to the implementation of support for veterinary practitioners in relation to adverse events were identified during thematic analysis of the comments (i) The importance of feasibility (ii) The need for a multifaceted and flexible approach, and (iii) The influence of human thought and behaviour. The entire list of free text comments received in each round can be found at S3 File. The themes are discussed along with exemplar quotes below.

(i) The importance of feasibility

Many comments related to the degree to which adverse event support could be practically achieved. ***Economic considerations*** were mentioned in terms of time constraints, financial efficiency and a reluctance to pass on costs to clients. Some voiced that provision of adverse event support for practitioners could not take precedent over clinical work, alluding to a need to focus efforts on the welfare of patients and the needs of clients above and beyond those of veterinary personnel.

*“The demand for meetings within the practice day is becoming unsustainable. We cannot have meetings and do clinical work at the same time. Scheduling time to perform these is difficult and whilst every attempt to have these meetings is made the great practical difficulty is having them when the clinical work does and needs to take precedent. All non-clinical time in practice is a price driver and ultimately results in additional costs to clients so whilst in theory all of the above would seem like a good idea, they present real and genuine practical difficulties in general practice”*

Further to economic considerations, concerns regarding the influence that ***practice size and structure*** may have on the implementation of such recommendations were raised. Some highlighted the advantages that larger practices, particularly those with corporate investment, may have. However, some comments suggested that independent practice structures may be better placed to offer the kind of support suggested within the recommendations.

*…”.. in a corporate setting it may be more appropriate for these tasks to be taken on/supported by practice support teams at a central level. I’m not sure we would have managed most of these recommendations as an independent vet practice”*

(ii)  The need for a multifaceted and flexible approach

Comments relayed the importance of support that pays heed to the ***nuanced nature of adverse events,*** suggesting that recommendations should be flexible rather than overly prescriptive. Prescheduled vs reactive support in the aftermath of adverse events and an allusion that although protocols are important, recommendations may perhaps be best used as more flexible guidelines.

*“Whilst I think it’s really important to have established protocols in place, I also think it’s important to have a degree of flexibility so that a team can react to the situation in front of them. Not all adverse events need a debrief….”*

There was a nod by many to ensuring that support is ***inclusive and accessible***. Comments included discussion surrounding the diverse nature of veterinary care provision and needs of those involved in part-time, antisocial, lone, ambulatory, telecommunications and complex team working.

*“…just generally making sure that whatever type of work they’re [veterinary practitioners] in they know about it [the support] and are able to use it if they need”*

No matter what the type of work a practitioner was engaged in, their potentially differing emotional and psychosocial needs were also raised as a key consideration.

*“….[support] may overlook personality types of vets and clients, perhaps a bespoke approach to each situation may be preferable?”*

*“[support] needs to be inclusive of different personalities and neurodiversity, one approach may not work for all”*

(iii) The influence of human thought and behaviour

Creating support that enshrines a sense of ***psychological safety*** amongst practitioners was repeatedly referenced.

*“[support] is only ever going to be helpful if the environment is such that people feel able to talk openly, otherwise they can be very stressful”*

Getting ***‘buy-in’*** across all veterinary practice staff from leadership to frontline practitioners was deemed a key influencer on the success of implementation. A gradual introduction of measures, which could be used to sculpt and embed positive change was suggested.

*“…anything that is seen as unduly burdensome and inflexible is potentially not to happen or be sustained; and getting something going to build upon has to be the priority”*

While many saw benefit in ***training and education***, others were more sceptical of its importance and instead suggested that a positive practice culture, underpinned by strong role modelling from leadership personnel was most important.

## Discussion

To the authors knowledge this is the first time research has been conducted to produce an evidence-based veterinary specific framework that can be used when endeavouring to support practitioners in relation to adverse events. The recommendations are not only relevant to occupational stress mitigation but contribute to emerging evidence surrounding adverse event prevention techniques and the link between veterinary practitioner wellbeing, patient safety and quality improvement. The outcome of this research is particularly relevant to the veterinary profession as it is grounded in the opinions of a range of stakeholders, including practitioners themselves, alongside drawing from the available research evidence.

### The recommendations

The findings reflect an appetite for supporting veterinary practitioners in relation to their experiences of adverse events. A relatively high number of recommendations reached consensus overall, with almost two thirds of the initial recommendations doing so within the first round. The diverse final list of recommendations suggests that a broad range of psychological assistance and practical engagement interventions are perceived to be of potential benefit and reflects the complexity of supporting veterinary practitioners in relation to adverse events. Qualitative insights extrapolated in the study, further suggest that a ‘one size fits all’ approach would perhaps be futile. Adherence to perceived bureaucratic protocol risks inducing a cycle of fear and disengagement from veterinary practitioners in relation to their involvement in adverse events. Peer mediated support, built on a premise of mutual agreement of what is beneficial, has previously been suggested to be preferable and is supported by the findings of this study [34]. It will be useful to use the lens of House’s Social Support Theory [48] when designing and refining future peer mediated adverse event support interventions. It suggests that relationships are essential for wellbeing and that social connections can reduce stress because they allow sharing of emotional, informational, instrumental and appraisal support. For such social support to work, it must not only be available but have a structure and be deliverable within the specific context that it is intended [49].

Many of the recommendations reaching consensus relate to preventative or ‘primary’ measures that can be embedded within practice in an attempt to prevent adverse events from happening, as well as reducing the stressful impact when they do. This pays heed to the importance of promoting organisational safety culture [50], the relevance of which is recognised in the veterinary literature [51,52] but worthy of further consideration. Culture refers to shared values and beliefs and is posited to not only reflect human thought and behaviour, but to influence it [53]. As a component of organisational culture, safety culture is considered a measurable construct and an indicator of risk within the healthcare sector [54]. According to seminal safety literature, five interlinked domains contribute to a safety culture [55]; a safety culture is an informed, reporting, learning, flexible and just culture. This means that there is a collective awareness and willingness to be open and honest about risks and hazards, that efforts are made to mitigate such risks and that individuals who are involved are treated fairly in response to their involvement. Many of the recommendations produced as an output of this research broadly encapsulate and provide a practical means of enabling a veterinary safety culture as well as facilitating practitioner wellbeing.

Nearly a third of the recommendations reaching eventual consensus refer to education and training and education and training was identified as a subcategory influencing the implementation of the recommendations during the qualitative analysis. Educational support falls within the remit of professional skills, non-technical skills [56] or non-technical competencies [57] training, which is increasingly honoured for its role in enhancing patient outcomes and practitioner wellbeing [58]. Plentiful literature surrounding veterinary leadership [59–61], team work [62,63] and communication training exists. However, exploration into training that prepares veterinary practitioners to review adverse events, and to support themselves and colleagues who are emotionally affected, is lacking.

In contrast, there has been exploration of resilience to emotional distress in response to adverse events in the human medical profession [64]. Early research suggests that interventions aiming to enhance resilience of practitioners and students may be useful in reducing the detrimental impacts they suffer in relation to adverse events [65,66]. One way that practitioners may develop resilience is to use their understanding of situations and their associated emotional reactions to inform how they respond in similar events in the future. The sentiment is encapsulated within reflective practices. Individualistic reflective practice is promoted and assessed by the veterinary professions’ current regulatory body, the Royal College of Veterinary Surgeons’ (RCVS), within a professional key skills (PKS) module of a post graduate certificate and has shown promise in reducing veterinary practitioner stress [58]. Reflective practice is underpinned by the theory that future performance can be improved by analysing one’s own experiences rather than being directly taught. A number of the final recommendations in this study relate to collective reflective practice. Previous preliminary research has shown that group reflective practice may be beneficial in facilitating veterinary practitioners to process the emotional [67] and ethical aspects of veterinary care [68,69]. It would, however, also befit the profession to explore whether reflectively practicing in teams could more specifically mitigate the emotional impact suffered in response to adverse events. The findings in the present study highlight that there is a desire for training and education in relation to adverse events as well as reflective practice. Further research is needed to understand whether practitioners are better supported to emotionally learn from adverse events through direct teaching or via reflective processes or if there should ideally be a balance of the two approaches.

To date, there is a lack of peer-reviewed literature upholding the notion that adverse event review meetings within practice reduce stress experienced by veterinary practitioners [70]. Many of the recommendations reaching consensus specifically relate to the implementation of adverse event review processes. In addition to suggesting that adverse event review has a role to play in mitigating practitioners’ associated stress, it is hoped that this finding will stimulate the design and refinement of veterinary specific adverse event review processes, which has previously been called for in relation to improving veterinary patient safety [71]. Adverse event review meetings also provide an intuitive platform for signposting to other more specific emotional support services.

### The methodological process

The review of the literature and inclusion of suggestions from prior work with veterinary stakeholders was conducted as a means of screening articles pertaining to interventions that may be beneficial for veterinary practitioners in relation to adverse event avoidance and post-event support. The fact that a relatively high number of recommendations reached consensus for inclusion and none reached consensus for exclusion potentially reflects that the language, structure and content of the recommendations was suitable and that the process used to initially develop them was credible. Involving potential end users or beneficiaries of the recommendations in the processes was essential to ensure the outcome was fitness for purpose. Strength lies in the time and consideration taken in planning the panel composition. Including diverse and heterogenous stakeholder views [72], and having low panellist dropout rates [42] increases the validity of the results in consensus building exercises. Both were the case in this study. The purposive technique used to select the Delphi Panellists reduced research time and likely enhanced yield of appropriate and useful information. Including clients within the panel was particularly important in ensuring a more balanced and representative outcome [73]. The planned panel composition was achieved and the response rate was high throughout the rounds, yet bias introduced by such non-probability sampling is possibly a limitation in this study [74]. The results may have differed significantly had an alternative panel composition been used. As some of the panellists had partaken in interview and focus groups studies on the same topic prior to the e-Delphi study, a degree of selection bias may also have been at play. Although panellists were reassured that all responses would remain anonymous, pre-existing knowledge of the research and the researcher meant that there was a risk that they may have tailored their responses (i) in line with what they perceived would be a desirable research output or (ii) due to concern that they would be identified. The lack of panellist drop-out between the first two rounds and minimal drop out thereafter is unlikely to have affected the outcome, particularly as the panel composition remained balanced.

The e-Delphi consensus building approach, used to determine the recommendations most relevant to the veterinary profession, reduced the risk of the halo effect that can be imparted by professional and personal hierarchy [75]. By anonymising the responses and allowing panellists to reconsider stance based on knowledge of others’ responses, reflective thinking and sharing of honest opinion was promoted. Beyond the first round, the number of the initial recommendations reaching consensus remained unchanged. This finding could suggest two things. Firstly, it could be concluded that panellists did not commonly change their mind after a period of reflection and that the influence of other panellists’ responses was minimal. i.e., The bandwagon effect bias, where participants change responses to align with a majority view [76], did not appear to play a significant role in this study. Conversely, it could further highlight aforementioned limitations surrounding the panel composition and a potential lack of diverse thinking amongst respondents. Despite this, the high response rate, along with the positive free text feedback regarding the process and the recommendations, provides evidence of successful stakeholder engagement in the topic.

If the study was to be repeated, a consensus meeting event to encourage panellists to converse regarding their views throughout the process could be included. This could perhaps occur via an anonymised online discussion group and would allow misunderstandings and discrepancies to be discussed and enable formulation of more nuanced recommendations.

The large number of free text responses voluntarily offered by panellists highlights that they had many comments regarding the formulation and implementation of the recommendations. To enhance rigour, the research group employed a reflexive approach [47], and the primary researcher documented rationale for decisions surrounding panel composition, panellist selection, initial recommendation development and the thematic analysis process. Although analysis of the free text comments provided useful insight into factors that may influence implementation of support for veterinary practitioners in relation to their experiences of adverse events, the findings must be considered interpretative and cannot necessarily be generalised beyond the specific recommendations formulated.

### Stressful Adverse Veterinary Events Support (SAVES) Framework: implementing and assessing the impact of the recommendations

The desired aim of the framework produced in this study is to avoid or mitigate stress amongst veterinary practitioners in relation to adverse events and to improve patient safety. For the recommendations contained to contribute to this aim, they need to be translated into a format that is engaging and usable within the veterinary sector. This relies on identifying pathways to the uptake of the recommendations and designing a visually appealing and practically agreeable resource. Implementation occurs between the development and evaluation phases of occupational interventions, and is defined as the process which transforms working conditions to achieve as desired outcome within a specific organisational setting [77]. Implementation is suggested to be as, if not more, important to the success of interventions than the content. The themes generated as a result of the thematic analysis broadly align with the concepts of acceptability, fidelity and feasibility, which are suggested as central to the implementability of scalable and sustainable interventions [78]. The insights unveiled in this study suggest that the success of support that is implemented for veterinary practitioners in relation to adverse events relies on it being sensitive to the context of veterinary practices. Further work is therefore needed to produce a user-friendly and fit for purpose resource. This relies on assessing what successful implementations look like across specific practice settings and monitoring post-implementation outcomes.

## Conclusions

Adverse events cause harm to veterinary patients and can be stressful for practitioners who are involved. To improve both occupational mental health and patient safety, practitioners must have access to guidance that assists in the prevention of adverse events, mitigates any personal emotional detriment experienced in relation to the occurrence of an adverse event and facilitates reflective learning and improvement in the aftermath. This study is important because it generated the first veterinary specific evidence-based guidance for supporting practitioners in relation to preventing or dealing with adverse events. Further work is needed to assess firstly the implementability, and later the impact of, the recommendations contained. However, it is hoped that the framework will be received by the veterinary profession as a pragmatic stimulus for actioning and improving practical support, whilst driving further research in this much needed area.

## Supporting information

S1 File Complete results indicating levels of agreement with recommendations and in rounds 1, 2 and 3 and whether recommendations reached consensus for inclusion or were excluded from the final framework. (PDF)

S2 File Complete list of recommendations that were not included in the final framework. (PDF)

S3 File Complete list of free text comments. (PDF)
