# Supplementary material for: Using an e-Delphi consensus technique to develop the Stressful Adverse Veterinary Events Support (SAVES) Framework
Source: PLoS One. 2025 Jun 24;20(6):e0326222. doi: 10.1371/journal.pone.0326222 (PMC12186887; doi:10.1371/journal.pone.0326222)
Supplement: S1 File — (PDF) [file pone.0326222.s001.pdf]

Results round 1

**Recommendations for primary support – to provide a working environment that proactively addresses and prevents stress in veterinary practitioners before involvement in adverse events.**	**Number (percentage) of panellists indicating 6 or 7 on Likert scale**	**Number (percentage) of panellists indicating 1 or 2 on Likert scale**	**Number (percentage) of panellists indicating 3,4 or 5 on Likert scale**	**Consensus reached for inclusion (✓) or exclusion (×) at round 1 or consensus not reached (-) at round 1**
Veterinary practitioners should receive basic education/training in the causes and types of adverse event.	44/50 (88)	4/50 (8)	2/50 (4)	✓
Veterinary practitioners should receive basic education/training in adverse event review techniques.	44/50 (88)	4/50 (8)	2/50 (4)	✓
Veterinary practitioners should receive training in communicating with clients in the aftermath of an adverse event.	44/50 (88)	4/50 (8)	2/50 (4)	✓
Veterinary practitioners should receive education about veterinary regulation, governance and legal responsibilities in relation to adverse events.	38/50 (76)	4/50 (8)	8/50 (16)	✓
Veterinary practitioners should receive education/training about the emotional and professional impacts of adverse events.	38/50 (76)	4/50 (8)	8/50 (16)	✓
Veterinary practitioners should receive regular resilience training.	27/50 (54)	6/50 (12)	17/50 (34)	-
Regular veterinary practitioner group meetings, where clinical aspects of cases are discussed, should be conducted within practice.	42/50 (84)	3/50 (6)	5/50 (10)	✓
Regular veterinary practitioner group meetings, where the social, emotional and professional aspects of clinical work are discussed, should be conducted within practice.	32/50 (64)	3/50 (6)	15/50 (30)	-
Regular veterinary practitioner group meetings, where ethical aspects of clinical work are discussed, should be conducted within practice.	28/50 (56)	3/50 (6)	19/50 (38)	-
Veterinary practitioner group meetings should be compulsory to attend, or provision made for those unable to attend to be updated on the meeting discussions.	23/50 (46)	6/50 (12)	21/50 (42)	-
Veterinary practitioner group meetings should always be led by facilitators who are trained in conducting the specific meeting type.	18/50 (36)	3/50 (6)	29/50 (58)	-
Veterinary practitioner group meetings should always be prescheduled.	24/50 (48)	5/50 (10)	21/50 (42)	-
Veterinary practitioner group meetings should always be conducted within working hours.	15/50 (30)	5/50 (10)	30/50 (60)	-
Pre-scheduled meetings between one practitioner and one equally or more experienced practitioner, where any aspect of clinical work can be discussed, should be conducted within the practice.	17/50 (34)	4/50 (8)	29/50 (58)	-
Veterinary practitioners should have the ability to discuss any social, emotional, professional, ethical or clinical aspect of a case with an equally or more experienced practitioner on a one-to-one basis at any time.	32/50 (64)	5/50 (10)	13/50 (26)	-
Written comments/suggestions/concerns from veterinary practitioners about any social, emotional, professional, ethical or clinical aspects of cases should be encouraged through the provision of physical or virtual comment boxes within the practice.	28/50 (56)	4/50 (6)	19/50 (38)	-
Veterinary practitioners contributing written comments/suggestions/concerns regarding social, emotional, professional, ethical or clinical aspects of care via comment boxes should have the ability to remain anonymous.	35/50 (70)	4/50 (8)	11/50 (22)	-
Approaches used for reviewing and learning from adverse events should be pre-agreed by veterinary practitioners.	28/50 (56)	3/50 (6)	19/50 (38)	-
Designated roles and responsibilities for recording and reviewing adverse events should be pre-agreed by veterinary practitioners within a practice.	28/50 (56)	4/50 (8)	18/50 (34)	-
Approaches to responding to adverse events should be reviewed at predetermined time periods within a practice (e.g. quarterly, bi-annually, annually).	28/50 (56)	6/50 (12)	16/50 (32)	-
Determining what is acceptable and unacceptable veterinary practitioner behaviours surrounding adverse events and adverse event review should be pre-agreed by veterinary practitioners working within a practice.	24/50 (48)	5/50 (10)	21/50 (42)	-
Adverse event review processes, roles, responsibilities and expected conduct should be clearly documented and accessible for reference by veterinary practitioners.	38/50 (76)	3/50 (6)	9/50 (18)	✓

Veterinary practitioners' experiences should be used as feedback to regularly review processes, roles, responsibilities and conduct in relation to adverse event review.	39/50 (78)	3/50 (6)	8/50 (16)	✓
Veterinary practitioners should pre-agree designated role responsibilities for communicating with owners of animals affected by an adverse event within a practice.	26/50 (52)	4/50 (8)	20/50 (40)	-
A written policy or 'Charter' explaining the rights and responsibilities of both veterinary practitioners and veterinary clients in relation to adverse events should be clearly displayed within veterinary practices.	19/50 (38)	4/50 (8)	27/50 (54)	-
Information regarding professional and emotional support available from external bodies (e.g. professional body advice lines, BVA, VDS, Vetlife) should be clearly visible within veterinary practitioners' workplace environments.	38/50 (76)	2/50 (4)	10/50 (20)	✓

**Recommendations for secondary support – to provide a rapid and appropriate response in the aftermath of adverse events to mitigate the severity and duration of stress experienced by those involved.**	**Number (percentage) of panellists indicating 6 or 7 on Likert scale**	**Number (percentage) of panellists indicating 1 or 2 on Likert scale**	**Number (percentage) of panellists indicating 3,4 or 5 on Likert scale**	**Consensus reached for inclusion (✓) or exclusion (×) at round 1 or consensus not reached (-) at round 1**
Immediately following an adverse event, a one-to-one discussion between an involved veterinary practitioner(s) and a peer trained in Psychological First Aid (PFA) should take place to ensure the practitioner(s) is psychologically safe.	24/50 (48)	3/50 (6)	23/50 (46)	-
One-to-three weeks after an adverse event, a group discussion led by a peer trained in Critical Stress Incident Debriefing, comprising veterinary practitioners involved and those with knowledge of the event, should take place to facilitate reflection on both the emotional and clinical aspects of the event.	32/50 (64)	3/50 (6)	15/50 (30)	-
One week to several months after an adverse event, a group discussion led by a peer trained in Clinical Ethics Debriefing (CED), comprising veterinary practitioners involved and those with knowledge of the event, should take place to facilitate reflection on ethical aspects of the event.	22/50 (44)	3/50 (6)	25/50 (50)	-
At a mutually agreed time after the event, a one-to-one discussion between an individual involved in an adverse event and an experienced clinician should take place as part of the individual's personal and professional learning and development.	40/50 (80)	4/50 (8)	6/50 (12)	✓
Adverse events should be reviewed during prescheduled meetings which are conducted during working hours.	35/50 (70)	3/50 (6)	12/50 (24)	-
Adverse events should be reviewed using a standardised template (eg. those used in root cause analysis/fishbone diagram/Five Why's/Six Sigma etc.).	25/50 (50)	2/50 (4)	23/50 (46)	-
Adverse event review should be conducted within a meeting which is open to all veterinary practitioners regardless of their degree of involvement in or knowledge of the event.	25/50 (50)	5/50 (10)	20/50 (40)	-
Adverse event review meetings should be led by a trained facilitator.	25/50 (50)	3/50 (6)	22/50 (44)	-
Adverse event review findings, learning and action points should be recorded and stored in a form accessible for reference by veterinary practitioners within a practice.	33/50 (66)	4/50 (8)	13/50 (26)	-
Adverse event review findings should not be considered during performance review or practice disciplinary proceedings.	22/50 (44)	7/50 (14)	21/50 (42)	-
Veterinary practitioners who are involved in an adverse event should always be provided with clear information regarding review and/or investigation processes.	43/50 (86)	3/50 (6)	4/50 (8)	✓
Veterinary practitioners who are involved in an adverse event should always be provided with clear information about the potential emotional and professional impact.	38/50 (76)	2/50 (4)	10/50 (20)	✓
Veterinary practitioners involved in adverse events should always be signposted to specific emotional support providers (e.g. Vetlife, VetSupport).	40/50 (80)	3/50 (6)	7/50 (14)	✓
Veterinary practitioners involved in adverse events should always be signposted to professional body advice lines (e.g. VDS, BVA etc).	38/50 (76)	3/50 (6)	9/50 (18)	✓

**Recommendations for tertiary support – to provide remedial support for practitioners who experience severe and/or longer term personal and professional consequences in the aftermath of adverse events.**	**Number (percentage) of panellists indicating 6 or 7 on Likert scale**	**Number (percentage) of panellists indicating 1 or 2 on Likert scale**	**Number (percentage) of panellists indicating 3,4 or 5 on Likert scale**	**Consensus reached for inclusion (✓) or exclusion (×) at round 1 or consensus not reached (-) at round 1**
Veterinary practices should facilitate reasonable adjustments to duties that are requested by practitioners who are impacted emotionally and/or professionally by involvement in an adverse event.	31/50 (62)	3/50 (6)	16/50 (32)	-
Veterinary practices should facilitate provision of reasonable mentoring or additional training requests by practitioners who are impacted emotionally or professionally by involvement in an adverse event.	41/50 (82)	3/50 (6)	6/50 (12)	✓
Veterinary practices should regularly review the ongoing support needs of practitioners who are emotionally and/or professionally impacted by involvement in an adverse event.	41/50 (82)	3/50 (6)	12/50 (24)	✓
Veterinary practices should provide clear information to practitioners who are emotional and/or professionally impacted by involvement in an adverse event, regarding the availability of workplace programs that offer free and confidential assessments, short term counselling, referrals and follow-up (i.e. Employee Assistance Programs).	38/50 (76)	2/50 (4)	10/50 (20)	✓
Veterinary practitioners should be offered opportunity to contribute to practice discussions about their personal experiences of being involved in an adverse event.	44/50 (88)	2/50 (4)	4/50 (8)	✓
Veterinary practitioners should be offered opportunity to lead an improvement project based on lessons learnt from being involved in an adverse event.	39/50 (78)	2/50 (4)	9/50 (18)	✓

<u>Summary of results round 1</u>

- 46 recommendations entered round 1
- 19 recommendations reached consensus for inclusion in round 1
- 0 recommendations reached consensus for exclusion in round 1
- 27 recommendations did not reach consensus in round 1.
    - Based on free text comments and suggestions provided by panellists:
        - 10 recommendations remained unchanged.
        - 4 were formulated by amalgamating 9 recommendations into 4.
        - 8 were amended.
        - 3 new recommendations were formulated.
- 25 recommendations to enter round 2.

Results round 2 (*amended ^amalgamated &new recommendations following round 1)

Recommendations for primary support – to provide a working environment that proactively addresses and prevents stress in veterinary practitioners before involvement in adverse events.	Number (percentage) of panellists indicating 6 or 7 on Likert scale	Number (percentage) of panellists indicating 1 or 2 on Likert scale	Number (percentage) of panellists indicating 3,4 or 5 on Likert scale	Consensus reached for inclusion (✓) or exclusion (×) at round 2 or consensus not reached (-) at round 2
*Veterinary practitioners should receive training about strategies that veterinary teams may collectively use to withstand or recover from personal and professional impacts of adverse events (e.g. team resilience training).	32/49 (65.3)	2/49 (4.1)	15/49 (30.6)	-
&Veterinary practitioners should receive training in how to support colleagues who may be emotionally and/or professionally affected in the aftermath of an adverse event.	34/49 (69.3)	0/49 (0)	16/49 (32.6)	-
&Veterinary practitioners should receive training in how to communicate the risk of adverse events to clients.	38/49 (77.6)	1/49 (2)	10/49 (20.4)	✓
^Practitioners should be given the opportunity to attend veterinary practitioner group meetings where reflection and discussion of the non-clinical aspects (social, emotional, professional and ethical) of veterinary care is encouraged.	35/49 (71.4)	2/49 (4.1)	12/49 (24.1)	-
*Veterinary practitioners should be given the opportunity to attend group meetings, or provision made for them to receive an update on any meetings they do not attend.	28/49 (57.1)	4/49 (8.2)	17/49 (34.7)	-
*Veterinary practitioner group meetings should always be led by facilitators who are trained in conducting the specific meeting type.	33/49 (67.3)	1/49 (2)	15/49 (30.6)	-
*Veterinary practitioners should be notified in advance about the intended time, format and purpose of group meetings where clinical and non-clinical aspects of work are to be discussed.	38/49 (77.6)	2/49 (4.1)	9/49 (18.4)	✓
*Veterinary practitioner group meetings should be offered within working hours but may be conducted out of working hours if veterinary team members are in agreement.	38/49 (77.6)	3/49 (6.1)	8/49 (16.3)	✓
^One-to-one discussions about any clinical or non-clinical aspects of work (social, emotional, ethical, professional) should be encouraged via the provision of a 'buddy' system within practices.	28/49 (57)	3/49 (6.1)	18/49 (36.7)	-
Written comments/suggestions/concerns from veterinary practitioners about any social, emotional, professional, ethical or clinical aspects of cases should be encouraged through the provision of physical or virtual comment boxes within the practice.	25/49 (51)	4/49 (8.2)	20/49 (40.8)	-
Veterinary practitioners contributing written comments/suggestions/concerns regarding social, emotional, professional, ethical or clinical aspects of care via comment boxes should have the ability to remain anonymous.	33/49 (67.3)	4/49 (8.2)	12/49 (24.5)	-

^Adverse event review processes, roles, responsibilities and expected conduct should be pre-agreed by veterinary practitioners working within a practice.	36/49 (73.5)	2/49 (4)	11/49 (22.4)	-
Designated roles and responsibilities for recording and reviewing adverse events should be pre-agreed by veterinary practitioners within a practice.	34/49 (69.4)	14/49 (28.6)	18/50 (34)	-
*Adverse event review processes, roles, responsibilities and expected conduct should be reviewed at predetermined time periods within a practice (e.g. quarterly, bi-annually, annually).	31/49 (63.3)	15/49 (30.6)	15/49 (30.6)	-
Veterinary practitioners should pre-agree designated role responsibilities for communicating with owners of animals affected by an adverse event within a practice.	26/49 (53.1)	4/49 (8.2)	19/49 (38.8)	-
A written policy or 'Charter' explaining the rights and responsibilities of both veterinary practitioners and veterinary clients in relation to adverse events should be clearly displayed within veterinary practices.	22/49 (44.9)	5/49 (10.2)	22/49 (44.9)	-

**Recommendations for secondary support – to provide a rapid and appropriate response in the aftermath of adverse events to mitigate the severity and duration of stress experienced by those involved.**	**Number (percentage) of panellists indicating 6 or 7 on Likert scale**	**Number (percentage) of panellists indicating 1 or 2 on Likert scale**	**Number (percentage) of panellists indicating 3,4 or 5 on Likert scale**	**Consensus reached for inclusion (✓) or exclusion (×) at round 1 or consensus not reached (-) at round 2**
^Veterinary practitioners who are involved in an adverse event should be offered opportunity to discuss the non-clinical (social, emotional, ethical and professional) aspects of the event within a group meeting at mutually agreed times in the aftermath (group debriefing).	41/49 (83.7)	3/49 (6.1)	5/49 (10.2)	✓
Adverse events should be reviewed during prescheduled meetings which are conducted during working hours.	33/49 (67.3)	1/49 (2)	15/49 (30.6)	-
Adverse events should be reviewed using a standardised template (eg. those used in root cause analysis/fishbone diagram/Five Why's/Six Sigma etc.).	23/49 (46.9)	0 (0)	26/49 (53.1)	-
Adverse event review should be conducted within a meeting which is open to all veterinary practitioners regardless of their degree of involvement in or knowledge of the event.	28/49 (57.1)	4/49 (8.2)	17/49 (34.7)	-
Adverse event review meetings should be led by a trained facilitator.	29/49 (59.2)	0 (0)	20/49 (40.8)	-
*Adverse event review findings, learning and action points should be recorded and stored in an anonymised secure form for reference by veterinary practitioners within a practice.	39/49 (79.6)	3/49 (6.1)	7/49 (14.3)	✓
Adverse event review findings should not be considered during performance review or practice disciplinary proceedings.	26/49 (53.1)	2/49 (4)	21/49 (42.9)	-
[&]Veterinary practitioners should be encouraged to enter details of adverse events on a centralised reporting system (such as VetSafe; VDS) and use this to review adverse events quarterly.	35/49 (71.4)	0 (0)	14 (28.6)	-

**Recommendations for tertiary support – to provide remedial support for practitioners who experience severe and/or longer term personal and professional consequences in the aftermath of adverse events.**	**Number (percentage) of panellists indicating 6 or 7 on Likert scale**	**Number (percentage) of panellists indicating 1 or 2 on Likert scale**	**Number (percentage) of panellists indicating 3,4 or 5 on Likert scale**	**Consensus reached for inclusion (✓) or exclusion (×) at round 1 or consensus not reached (-) at round 2**
Veterinary practices should facilitate reasonable adjustments to duties that are requested by practitioners who are impacted emotionally and/or professionally by involvement in an adverse event.	31/49 (63.3)	2/49 (4)	16/49 (32.7)	-

Summary of round 2

- 25 recommendations entered round 2
- 5 recommendations reached consensus for inclusion during round 2
- 0 recommendations reached consensus for exclusion during round 2
- 20 recommendations remained to enter round 3

Results round 3 (*amended ^amalgamated &new recommendations following round 1)

Recommendations for primary support – to provide a working environment that proactively addresses and prevents stress in veterinary practitioners before involvement in adverse events.	Number (percentage) of panellists indicating 6 or 7 on Likert scale	Number (percentage) of panellists indicating 1 or 2 on Likert scale	Number (percentage) of panellists indicating 3,4 or 5 on Likert scale	Consensus reached for inclusion (✓) or exclusion (×) at round 2 or consensus not reached (-) at round 3
*Veterinary practitioners should receive training about strategies that veterinary teams may collectively use to withstand or recover from personal and professional impacts of adverse events (e.g. team resilience training).	34/44 (77.3)	0/44 (0.0)	10/44 (22.7)	✓
&Veterinary practitioners should receive training in how to support colleagues who may be emotionally and/or professionally affected in the aftermath of an adverse event.	33/44 (75)	0/44 (0.0)	11/44 (25)	✓
^Practitioners should be given the opportunity to attend veterinary practitioner group meetings where reflection and discussion of the non-clinical aspects (social, emotional, professional and ethical) of veterinary care is encouraged.	33/44 (75)	1/44 (2.3)	10/44 (22.7)	✓
*Veterinary practitioners should be given the opportunity to attend group meetings, or provision made for them to receive an update on any meetings they do not attend.	26/44 (59.1)	3/44 (6.8)	15/44 (34.1)	-
*Veterinary practitioner group meetings should always be led by facilitators who are trained in conducting the specific meeting type.	24/44 (54.5)	0/44 (0.0)	20/44 (42.4)	-
^One-to-one discussions about any clinical or non-clinical aspects of work (social, emotional, ethical, professional) should be encouraged via the provision of a 'buddy' system within practices.	21/44 (47.7)	1/44 (2.3)	22/44 (50)	-
Written comments/suggestions/concerns from veterinary practitioners about any social, emotional, professional, ethical or clinical aspects of cases should be encouraged through the provision of physical or virtual comment boxes within the practice.	17/44 (38.6)	3/44 (6.8)	24/44 (54.5)	-
Veterinary practitioners contributing written comments/suggestions/concerns regarding social, emotional, professional, ethical or clinical aspects of care via comment boxes should have the ability to remain anonymous.	28/44 (63.6)	2/44 (4.6)	14/44 (31.8)	-
^Adverse event review processes, roles, responsibilities and expected conduct should be pre-agreed by veterinary practitioners working within a practice.	32/44 (72.7)	0/44 (0.0)	12/44 (27.3)	-
Designated roles and responsibilities for recording and reviewing adverse events should be pre-agreed by veterinary practitioners within a practice.	24/44 (54.5)	0/44 (0.0)	20/44 (45.5)	-
*Adverse event review processes, roles, responsibilities and expected conduct should be reviewed at predetermined time periods within a practice (e.g. quarterly, bi-annually, annually).	28/44 (63.4)	2/44 (4.5)	14/44 (31.8)	-
Veterinary practitioners should pre-agree designated role responsibilities for communicating with owners of animals affected by an adverse event within a practice.	14/44 (31.8)	3/44 (6.8)	27/44 (61.3)	-
A written policy or 'Charter' explaining the rights and responsibilities of both veterinary practitioners and veterinary clients in relation to adverse events should be clearly displayed within veterinary practices.	13/44 (29.5)	3/44 (6.8)	28/44 (63.6)	-

**Recommendations for secondary support – to provide a rapid and appropriate response in the aftermath of adverse events to mitigate the severity and duration of stress experienced by those involved.**	**Number (percentage) of panellists indicating 6 or 7 on Likert scale**	**Number (percentage) of panellists indicating 1 or 2 on Likert scale**	**Number (percentage) of panellists indicating 3,4 or 5 on Likert scale**	**Consensus reached for inclusion (✓) or exclusion (×) at round 1 or consensus not reached (-) at round 3**
^Adverse events should be reviewed during prescheduled meetings which are conducted during working hours.	37/44 (84)	0/44 (0.0)	7/44 (15.9)	✓
Adverse events should be reviewed using a standardised template (eg. those used in root cause analysis/fishbone diagram/Five Why's/Six Sigma etc.).	23/44 (52.2)	1/44 (2.3)	20/44 (45.5)	-
Adverse event review should be conducted within a meeting which is open to all veterinary practitioners regardless of their degree of involvement in or knowledge of the event.	27/44 (61.4)	2/44 (4.6)	15/44 (34.0)	-
Adverse event review meetings should be led by a trained facilitator.	21/44 (47.7)	2/44 (4.5)	21/44 (47.7)	-
Adverse event review findings should not be considered during performance review or practice disciplinary proceedings.	20/44 (45.5)	3/44 (6.8)	21/44 (47.7)	-
[&]Veterinary practitioners should be encouraged to enter details of adverse events on a centralised reporting system (such as VetSafe; VDS) and use this to review adverse events quarterly.	35/44 (79.5)	0/44 (0.0)	9/44 (20.4)	✓

**Recommendations for tertiary support – to provide remedial support for practitioners who experience severe and/or longer term personal and professional consequences in the aftermath of adverse events.**	**Number (percentage) of panellists indicating 6 or 7 on Likert scale**	**Number (percentage) of panellists indicating 1 or 2 on Likert scale**	**Number (percentage) of panellists indicating 3,4 or 5 on Likert scale**	**Consensus reached for inclusion (✓) or exclusion (×) at round 1 or consensus not reached (-) at round 3**
Veterinary practices should facilitate reasonable adjustments to duties that are requested by practitioners who are impacted emotionally and/or professionally by involvement in an adverse event.	30/44 (68.2)	1/44 (2.3)	13/44 (29.5)	-

<u>Summary of round 3</u>

- 20 recommendations entered round 3.
- 5 additional recommendations reached consensus for inclusion during round 3.
- 0 recommendations reached consensus for exclusion during round 3.
- 15 recommendations were automatically excluded as no consensus was reached.
