# Supplementary material for: Using an e-Delphi consensus technique to develop the Stressful Adverse Veterinary Events Support (SAVES) Framework
Source: PLoS One. 2025 Jun 24;20(6):e0326222. doi: 10.1371/journal.pone.0326222 (PMC12186887; doi:10.1371/journal.pone.0326222)
Supplement: S2 File — (PDF) [file pone.0326222.s002.pdf]

Recommendations that DID NOT reach consensus and were automatically excluded at the end of the study (n=15)

Veterinary practitioners should be given the opportunity to attend group meetings, or provision made for them to receive an update on any meetings they do not attend.

Veterinary practitioner group meetings should always be led by facilitators who are trained in conducting the specific meeting type.

One-to-one discussions about any clinical or non-clinical aspects of work (social, emotional, ethical, professional) should be encouraged via the provision of a ‘buddy’ system within practices.

Written comments/suggestions/concerns from veterinary practitioners about any social, emotional, professional, ethical or clinical aspects of cases should be encouraged through the provision of physical or virtual comment boxes within the practice.

Veterinary practitioners contributing written comments/suggestions/concerns regarding social, emotional, professional, ethical or clinical aspects of care via comment boxes should have the ability to remain anonymous.

Adverse event review processes, roles, responsibilities and expected conduct should be pre-agreed by veterinary practitioners working within a practice.

Designated roles and responsibilities for recording and reviewing adverse events should be pre-agreed by veterinary practitioners within a practice.

Adverse event review processes, roles, responsibilities and expected conduct should be reviewed at predetermined time periods within a practice (e.g. quarterly, bi-annually, annually).

Veterinary practitioners should pre-agree designated role responsibilities for communicating with owners of animals affected by an adverse event within a practice.

A written policy or 'Charter' explaining the rights and responsibilities of both veterinary practitioners and veterinary clients in relation to adverse events should be clearly displayed within veterinary practices.

Adverse events should be reviewed using a standardised template (e.g. those used in root cause analysis/fishbone diagram/Five Why's/Six Sigma etc.).

Adverse event review should be conducted within a meeting which is open to all veterinary practitioners regardless of their degree of involvement in or knowledge of the event.

Adverse event review meetings should be led by a trained facilitator.

Adverse event review findings should not be considered during performance review or practice disciplinary proceedings.

Veterinary practices should facilitate reasonable adjustments to duties that are requested by practitioners who are impacted emotionally and/or professionally by involvement in an adverse event.
