# Supplementary material for: Using an e-Delphi consensus technique to develop the Stressful Adverse Veterinary Events Support (SAVES) Framework
Source: PLoS One. 2025 Jun 24;20(6):e0326222. doi: 10.1371/journal.pone.0326222 (PMC12186887; doi:10.1371/journal.pone.0326222)
Supplement: S3 File — (PDF) [file pone.0326222.s003.pdf]

Round 1

These should in my opinion be regular and formal, which in reality means it will actually happen, it may feel unnecessary until you really need it, and you cannot predict this, thus making it a normal part of being in clinical practice is essential.

resilience training needs definition

There is a balance required between appropriate education without triggering defensive practice that has yet to be found

Despite (generally) only senior staff/management/one designated staff member actively reviewing adverse events, if training about the review techniques and their purpose isn't provided to the whole team engagement and reporting of adverse events will be low and so the techniques won't be as effective. Resilience training is important, but I fear that people switch off and disengage if it is delivered too frequently/incorrectly. I thinking resilience training needs to be specific to an event/moment rather than just a person. If the training is routine and regular it should be optional.

Resilience is important and training to help someone to operate at a sustainable level and thrive - tips on how to do that when under pressure is a good idea however Resilience as a term can sometimes still have negative connotations- such that the individual is faulty and needs training to sustain themselves perhaps in the face of poor systems, unsupportive environments and normal life Fand adverse events (not always possible- we are human)

I agree that professionals should receive education about the professional impact of adverse effects. Possibly emotional impact too, but individuals' reactions vary hugely so this would be difficult and have to be careful not to inadvertently cause fear. I think this depends on how it is structured and delivers- resilience training to chronically under-resourced teams can be very damaging.

As a medical practitioner I feel that training in dealing with difficult and emotional is pivotal.

All of these are essential. However, there is no 'single one-way suits all' method, so this should be borne in mind

I currently feel that further resilience training may be perceived to further stretch the capacity of practitioners and so have marked this lower to illustrate that

This used to be provided informally via non corporate clinic structures, didn't it?

Resilience training sounds interesting.

It would be helpful to have a better understanding of what 'resilience training' involves. I wonder whether a focus on resilience in this context shifts responsibility onto the individual in a way that is not always helpful?

resilience training places a lot of emphasis on individuals, however the problem is not usually the individual but the environment around them; even the most resilient people are not invincible; everyone needs infrastructure/support around them and emphasising resilience training can make people think it is a personal failing, when usually it isn't.

Resilience training is needed before commencing any veterinary training

resilience? too general. lots of people talking about it but I'm not sure you can educate on it. you can be as resilient as you want but sometimes things are just hard

Training on how to communicate with clients and peers following an adverse event can only be a helpful thing so that learning can happen and honesty can be used to provide a positive outcome

Adverse events are often overlooked in all aspects of veterinary medicine but they can be a reason for complaints for should be discussed openly and training on how to handle those situations should be provided

Is training the answer to this? Is there evidence training would improve outcomes for these - particularly in resilience there is very mixed/poor evidence of benefit from training

None of this was taught when I studied and most was learnt when I had been in practice more than 15 years and human factors motivated me to progress. Most of my new/recent graduate experiences of SAEs involved poor protocols and communication by senior vets, but for the first 10-15 years of my career I placed minimal emphasis on preparation and management of these and instead focused on improving my clinical performance to try to prevent them. I think mindset needs to change throughout the veterinary profession

caveat to above comment is that if including resilience training, we could also be providing training on systems and how to improve those, make them more robust, review and update to take some of the emphasis off the individual. Human factors training- recognition of own inherent limitations

Practitioners should be educated in adverse events which have occurred to others or be involved in sharing learns from adverse events

I think it would be helpful if veterinary practitioners received education and training in communicating risk so that they feel able to discuss risks of adverse events with owners before they occur

Regular updates throughout career as refresher/renew thinking should be highly recommended

Veterinary Practitioners should receive information about the resources available to help guide them through processing adverse events that could cause an emotional toll (this might be encompassed by one of the above already)

Those in a leadership role should be more strongly encouraged to

Perhaps inclusion of training in how to support colleagues going through the aftermath of an adverse event.

availability of CPD in these areas would be beneficial. Perhaps including the use of actors and role play scenarios

I think all of these should be offered/advisable to those in leadership/management roles in practice

we should be taught more about spectrum of care

This expectation puts a lot of pressure on the more experienced colleague, who may not be able to cope with this level of support. Who provides support to the most senior vet?

General comment. The demand for meetings within the practice day is becoming unsustainable. We cannot have meetings and do clinical work at the same time. Scheduling time to perform these is difficult and whilst every attempt to have these meetings is made the great practical difficulty is having them when the clinical work does and needs to take precedent. All non-clinical time in practice is a price driver and ultimately results in additional costs to clients so whilst in theory all of the above would seem like a good idea, they present real and genuine practical difficulties in general practice.

is complicated! Also 1:1 are not feasible in sole charge practice?

Depends on the meeting type and the nature of the case, it might not be appropriate for certain characters to remain in the room during de-brief (to protect culture/effectiveness). 20. yes an agreed time frame to review most near misses is required, but with an option of doing one sooner (immediately) if a obvious and potentially catastrophic near miss/adverse event happened that has caused concern in the team.

I agree these should be discussed but it may be there is an alternative space to discuss them not in the practice which may suit that group, I would sooner see a practice meet out of the practice to discuss than not meet at all. Small practices may operate differently to large practices.

G N MDT and Clinical meetings are the underpinning of modern medicine and this shouldn't be a question for veterinary medicine.

I think there are some good ideas in here but I worry that by being too prescriptive about how things should be done can be counter-productive and risk shifting the focus to processes rather than developing a culture in which these processes can happen naturally

Do not think communication with owners should always fall to particular person, sometimes discussion better between original clinician and client (24)

I find it hard to agree to young vets ever being required to participate in compulsory meetings due to the culture of bullying by older colleagues

This is a massive section! All these ideas COULD work in a large practice but in reality, very hard in small practices where it is potentially even more valuable. Also, there is not a one size fits all for the infinite number of variable issues

A general comment - proposals are sensible and while i agree that a schedule and structure is important to get the habit going anything that is seen as unduly burdensome and inflexible is potentially not to happen or be sustained; and getting something going to build upon has to be the priority

I do not believe meeting should be compulsory. Doing so may compromise individuals who do not have the capacity at a given time and may impact adversely on their emotional and mental wellbeing

I think this is too rigid, and may overlook personality types of vets and clients, perhaps a bespoke approach to each situation may be preferable?

I'd hope that anyone graduating with mrcvs understands ethics and no meetings should be necessary. 14- not sure i understand the reason behind this question. 18, 19 - vet are not the specialists in this area to pre-agree on how to deal with these situations, but yes need buy-in

Many of the above recommendations refer to pre-agreed or pre-scheduled systems and processes. Whilst I think it's really important to have established protocols in place, I also think it's important to have a degree of flexibility so that a team can react to the situation in front of them. Not all adverse events need a debrief. e.g. hot and cold debriefs. Hot debriefs will not be scheduled in advance. Each situation is unique and therefore protocols need to be suitably nuanced to reflect this. E.g. determining which individual calls an owner to report an adverse event - this will depend heavily on the individuals involved, their emotional response to the situation and how they personally wish to address the situation. This will vary for different individuals e.g. some would wish to take ownership of the situation and speak to the owner themselves; others may find that too distressing and prefer a senior manager to handle the communication. The same individual on different days may find their preferences change, depending on what else is going on at the time i.e. how full their bucket is, what they feel they can cope with. I'm not sure how easy it is to talk about the emotional aspects of an adverse event in a group setting. Perhaps these conversations are better had between two individuals. I don't think this necessarily needs to be limited to employee-line manager - sometimes having conversations outside of this formal/hierarchical relationship can be more helpful. I also don't think that person necessarily needs to have the same or more clinical experience to make the conversation valuable.

only ever going to be helpful if the environment is such that people feel able to talk openly, otherwise they can be very stressful. Talking within practice doesn't always help; many won't share how they feel in front of their colleagues; others find such meetings shaming, so it does depend on the practice environment and if that is wrong, then these meetings could be harmful, rather than helpful.

the equally or more experienced colleague needs to be willing, able and trained in the art of feedback and active listening

i think this will depend on the individual situation

In general practice - time availability could be a major hindrance to achieving some of these recommendations (20-21) and in a corporate setting it may be more appropriate for these tasks to be taken on/supported by practice support teams at a central level. I'm not sure we would have managed most of these recommendations as an independent vet practice.

19 - were roles/responsibilities to be assigned/agreed then it potentially becomes an issue when people are off - responsibility needs to be shared across all practice team members ideally

I think most vets are aware of who they can ask for help...I think the problem is when they don't feel supported by their actual practice/boss etc?

'in the practice", for us it would be difficult to have a private space in our building for such a meeting, so we might by choice not hold such a meeting "in the practice"

this might not always be possible and for some things it might not be needed. vets can lead discussions about vet things. 12. might need to have an unscheduled meeting if an emergency. 13. it can be more relaxed not in working hours if people are happy to do it but needs to be on a practice-by-practice basis as some will mind and some would prefer out of working time discussions.

Regular meetings where cases are discussed are very important. They should have a structure and be lead by a vet trained to mediate these meetings. A space for individuals to express their feelings is important as some personalities may be too shy or sensitive to discuss things openly especially in front of figures who may be more intimidating or judgemental.

so many of these depend on context - in a workplace with bullying or poor leadership some of these could be of no benefit/could become part of harm

An external regular person such as a counsellor should be employed to regularly meet eg monthly or 3 monthly individual practitioners to discuss anything they are struggling with emotionally, this may or may not be work related as it will all be impacting work for that individual

No specific recommendation rather just a nod to making any actions accessible e.g to vets who work part time. If meetings are the same time and scheduled always there is a risk some personnel will always miss the meeting, flexibility to suit each team means there is a greater chance of being inclusive

Introduction of preventative measures for adverse events ie training in closed communication loops, introduction of surgical safety checklists, regular cpr training, culture where staff feel able to seek help or advice if unsure, consent forms detailing risks of procedures clearly so informed consent from clients

In any one-to-one meetings young vets to be given sufficient notice of meeting and to be allowed to take someone of their choice to the meeting

Encouraging all staff to use Vetsafe reporting system, and review events from here quarterly.

Whilst I agree in principle with having robust, clear policies and procedures in place for handling an adverse event, by having these written and documented as a formal instruction/guidance might not allow flexibility to adapt the practitioners' or practice's response/approach to handling an individual circumstance. Whilst there might be common themes to many adverse events in veterinary practice, the circumstances are frequently nuanced.

Those in a leadership position should be more strongly encouraged to receive education/training in adverse event review techniques.

needs to exist - in practice ToB - not necessarily in clear sight unless better client education is required

I worry about the time frame being too long. Personally I think being able to move things to the back of your mind is important. As someone prone to negative thought on things it takes a great deal of effort to put things behind me. Dragging them out again having just packed them away seems counterproductive - to the mental state. Possible quite productive from a patient care perspective.

I am not sure about the feasibility of insisting on only specially trained practitioners running meetings, I think it is better for regular meetings with line management rather than requiring additional training. Resources such as vetsafe make the process very easy so I'm not sure what benefit the additional training will bring

depends on the nature of the incident.

access to a professionally trained peer may not be practicable for many practices and the appropriateness of such an approach would depend on the nature of the adverse event and may not be relevant for all situations

I don't know enough about the evidence/if there is evidence about an immediate debrief but I believe there is evidence that immediate counselling after a trauma is not necessarily helpful/encouraged. I would change it to ensure that the option for support/debrief is available immediately - an emotional check in rather than any in depth discussion about the case. it can never be wrong to be kind to someone but I have doubts about an immediate debrief. I like the idea of a mutually agreed time in 30. for 28 and 29 I do support a team debrief and having someone with expertise would be helpful- having this as a standard structured event would be helpful but I support careful case selection rather than a blanket rule to go through all cases in a group setting. There are some individuals who may be too vulnerable to be exposed to a group discussion and some cases may be better handled sensitively- not sure how you word that but what I am leaning to guidance vs hard and fast rule

In an ideal world this would occur immediately, but it unlikely to be feasible for those working in small teams/sole charge

Adverse events allow for learning in medicine and should be discussed as CPD for all staff.

Again, these are good ideas but the need for different types of training for debriefing may be off putting / impossible in all but the largest practices.

Often time of high emotion and do not think this is best time to force discussion, not everyone is comfortable discussing their emotions with colleagues and better to cool down before reflect - conversation immediately after event could make practitioner feel blamed/judged

I have concern about 1:1 meeting in case the vet involved is not comfortable with the designated one other person

all are different and possible risk of escalating the trauma if done immediately. 28: 1-3 weeks allows the initial high emotion to settle 29 and 30: should be risk based as re-opening a scar may not be helpful to some

An experienced clinician, may not always be the person with the most approachable manner and the emotional intelligence to feel psychologically safe to have these discussions

Have concerns about implementation of (mandatory?) training to be qualified in PFA, and would hope that any more senior colleague would be able to provide adequate support

Might this run the risk of re-opening old wounds that could be healing? I'm no psychologist and wouldn't begin to suggest I know or don't know. Just my thoughts.

All numbers: These are more challenging to achieve in small workplaces.

These ideas sound very good but I'm not sure how they would work in practice or how effective they would be at relieving the stress in a real-life situation.

I worry that some of these suggestions could make the individual feel worse; I know it would me, and repeated discussion about the event weeks/months later can bring back the trauma.

I think the content of this could be discussed at the same time as Q28

recommendation number 30 appears much more achievable in the general practice setting. 27-29...these would need to be outsourced in most practices

perhaps this should be offered but not enforced? I think the PFA should be external to the practice or the corporate body involved, ie: not assigned by head office/"in their pocket"

Whilst agree this should be aimed for, not sure how many practices actually have somebody trained in PFA. 2.28, quite a short interval, and the practitioner affected May potentially still be off work if very stressed

some of this could be done at the same time rather than having separate meetings. a one-one after the event and then a group meeting later down the line.

I feel a discussion near to the adverse event is more helpful for everyone to let out their feeling. The group leader may not be trained but does need to understand the point of the meeting that it is to learn and not lay blame. This factor is key to the discussion.

All points are very important

immediate psychological debriefing is contraindicated and should only be done within the evidence base - it's against NICE guidance on PTSD

Teach more human factors at university

Immediately after an incident a hot debrief should be held with all of those directly involved in the event, all should have training in how to do a hot debrief but not specific trained member of staff needs to be present, just those involved. A part of this hot debrief can be to ask if anyone would like a cold debrief later.

Perhaps inclusion of reference to other support- signposting as part of debrief- vet life, friends, family etc. I wonder if "safety planning" could form part of the immediate emotional support -that someone in the practice has an understanding that an individual involved in an event may feel vulnerable- they may already have vulnerabilities (mental health) and checking in with them before they go home- e.g. do they live alone? could they be with family or or friends that night?, how do they seem-plan through what their plan to do is if they start to feel upset later in the evening- call someone for support, personal care e.g. eat food, bath, exercise or just rest. Having a plan for someone so they feel safe immediately after an event and in the aftermath?

How would training be provided for those named roles above?

Following an adverse event, practitioners should have access to independent emotional support outside of their workplace.

I think there need to be suitable support measures that can be implemented in small practice teams without access to all these trained practitioners

Should have ability to go home/not have to go immediately back into consulting etc after adverse event when shaken/upset

Signposting and knowledge for the individuals involved for where to go and who to seek support from at any time - rather than only pre-determined and pre-defined meetings/event reviews

this may not always be needed, and if there is no fall out with the clients involved or any psychological stress involved to the team then perhaps shouldn't be dragged up again months down the line? Manage on a case-by-case basis.

more peer support and routine support, less 'first aid' 'psychological debriefing' immediate response which may be c/i if potential trauma

If in working hours some individuals will not be able to join in as emergency services at least will need to be provided. Finding time outside of work where there are no other distractions may be beneficial?

I believe this is important to facilitate honest reflection. It's important that your thoughts cannot be used as a stick to beat you. Particularly as those thoughts maybe half formed an emotionally weighted. 34 trained and regularly refreshed facilitator important. This is a form of counselling. Poorly handled counselling can be damaging. 33 not open to all.

I'm not sure that allowing all colleagues to access the review of the SAE will encourage engagement in the process when errors are made

does this include support staff or just vets? 36. As a general rule yes, very strongly agree, so people continue to engage with it and not feel like it used against the - but it might be difficult to have this as a blanket rule if clear and undeniable negligence is identified that goes against the RCVS code of conduct.

yes, I think this will help to normalise talking about adverse events and if everyone can be present to observe the issues affecting both them and their peers it is levelling - however I think case selection for the meetings is still critical. I can envisage this to be highly distressing for some individual vets if it was a particularly challenging case. 34- ideal world yes definitely - real world - the level and standard of training someone may have had will vary - with careful case selection and a compassionate attitude I think that practice teams can learn to do this by doing and the profession should embrace these event reviews- I would worry people may see "trained facilitator" as a barrier not to get on board and do these meetings - there is also the risk of perfectionism creeping in- people feeling they are not trained enough to start doing these thus missing out on even basic emotional support it could extend to practice team members. 35 - would agree with this if it included reference to "secure" method of storing action points and learnings (sadly need to consider malicious intent within practice and also limit risk of having to hand over in Subject Access Request if any identifying features of a client/case)

If this individual was someone trained in practice, then this is easy to facilitate. If this is an external person then it would be harder to organise. 36. If adverse events formed a pattern to an individual's behaviour in practice then this would cause a concern over their performance, so then it is relevant to discuss this in a performance review.

I think this could be detrimental to an individual in certain circumstances eg. serious professional misconduct. Q36- it may or may not be appropriate to involve these- I'm not sure how they can not be included if there is refusal to change behaviour and repeat incidents following a review.

the format for the review findings should also be anonymised so as to be GDPR compliant

Depends on contributing factors to adverse event

I feel concern about any number of colleagues being able to get involved as I think the vet involved may prefer some privacy

Again, potential issues in smaller practices. Re 36: adverse event review during performance pr practice disciplinary... repeated mistakes not learnt from could be justified, so blanket approach maybe not ideal

I think aspects of the adverse event review findings should/could be considered during performance review as this could highlight whether a veterinary colleague involved in the event participated in the

review process, whether they demonstrated an effort to learn from the event, or whether there is a pattern of behaviour that could constitute a concern to patient or human safety, etc...

May be preferable to ask if the person/s involved are comfortable with everyone else being present at the meeting? 36. I think that would depend how many adverse events have happened with any individual, if excessive then ought to be included.

Absolutely - for an isolated incident, but if one individual is repeatedly involved in adverse events, this may need to be considered for their own professional and personal performance/development

The challenge is providing trained facilitators in small workplaces

I think it's fine to include adverse event reviews in pre-scheduled veterinary practitioner meetings but I don't think they should be discussed exclusively within these meetings. Some situations require rapid debrief and we shouldn't expect the individuals involved to have to wait for a prescheduled meeting. An initial review/debrief should happen promptly. A lengthier review may be able to wait until a prescheduled meeting. I also think the sensitivity of the situation will determine who should/shouldn't be involved in the review process. This will vary significantly with each event and also with the individuals involved and how they are responding/coping with the aftermath. Whilst I think standardised templates can be really helpful, I also recognise that these types of conversation can feel very intimidating and hostile to some individuals. Sometimes the inclusion of these sorts of techniques can feel very unfamiliar and inorganic, which may increase an individual's level of anxiety around having the conversation. I think it's very important to maintain a strong level of empathy & compassion and not make the whole process too 'clinical''.

depending on the nature of the adverse event these may need to be scheduled at a non-pre-determined time. 32 - don't understand what these are! 33 - depending on the nature of the adverse event it may not be appropriate to include staff that were not involved.

although this would seem logical and more likely to contribute to psychological safety within teams - there might be instances where it was appropriate to include these findings (gross professional misconduct/negligence) especially if a pattern developed where repeated errors were being made or no improvements implemented

ideal but not essential, could be done outside of work if agreed by both parties?

agree they should be led by a trained facilitator, but if one not available, still important they happen

agree with this but those not involved should be managed so that they are not judgemental if they do not know all the facts

The team should be encouraged to develop resources that work best for them and their practice, this may involve drawing on standardised templated listed but it is more important that they work for the practice rather than comply to a previously published format

I worry that bringing up details of the potential professional impact would increase the degree of anxiety.

Impact to who? the patient, the owner, the staff involved or all of these? If someone is ignoring the event and pretending it didn't happen then all of these would be appropriate and necessary so they can realise the significance of the incident. However, if a staff member is visibly upset and blaming themselves for an adverse event, it might not be appropriate to have to inform them of the impact the adverse event will have on the owner, but it would be appropriate to talk about the effect it is having or might have on themselves e.g. validifying their feelings.

where appropriate - if there is no likelihood of review/investigation then no need to warn, 38- yes where appropriate - I suspect it would not be practical to do this for relatively minor adverse events and support must be sincere and meaningful

In all cases I would change the wording from "veterinary practitioners involved in adverse events" to all veterinary practitioners - as this information should be available to all so they understand and accept what will happen if an adverse event occurs, and this information is not just provided after it happens

I would hate the increase in frequency of signposting to reduce impact so people become word blind to them. Not all adverse events are equally serious and minor ones have less impact but should not be ignored

I think information about where support is accessible is useful and should be available. Need to be careful to keep it in proportion to the event, and if support channels are pushed too much it may end up being detrimental/adding to stress

Quite difficult to say "always" when there is such a range of adverse events

I'm not sure you can ever be fully prepared it the worst is to happen.

Not all people will react to being involved in an adverse event the same and obviously some adverse events have bigger impacts that others and so some of these suggestions need to be considered on a case-by-case basis.

None

None

case by case. not always needed

I think it depends on the situation as to whether a conservation requires direction to regulatory bodies or mental health support bodies and everyone reacts differently to different conversations.

On a more local level, knowing who they can turn to within their own practice environment may also be very helpful

None

These should will be difficult to provide by independent practice and if adopted may drive more practices into the corporate structure.

Within the practice will not always be the best place to support colleagues emotional needs, so recognising this and signposting to other supports can often best..

there could be merit in considering this to be an agreed process between the practitioner and practice to include discussion on how often check-ins happen, help to set expectations for all. It can be important for both the practice (the team) and the practitioner that reasonable adjustments do not necessarily become permanent unless mutually agreed - there can be impacts on the rest of the team/culture when adjustments are made for individual team members- can lead to problems it may also facilitate an extended period of helplessness? (just wondering)- that said any adjustments could well save the person's career and give them time when they might otherwise leave the profession. ideally helping practitioners move on and thrive would be preferable

Only if they were comfortable to do this.

This is valuable learning for all vets and may help drive changes to practice.

Do not think it is helpful to create environment where practitioner avoids the situation in which adverse event occurred eg no longer doing bitch spays - think prolongs emotional impact and perpetuates fear of mistake recurring

Agree with all these

This could be of benefit but I don't think the individual should be put under an pressure to do this before they feel ready or without the support they require.

None

Performing task again with mentor to rebuild confidence in ability useful

As a practice owner involved in a case I had no idea where to go for help for myself or my employees.

None

None

I think the main thing is to be open about why things happen. It can be hard to pick through everything but at least everyone learns. It's a tricky one though as many people will get defensive so training is important.

This is very relevant work

Round 2

I think if an adverse event happens, it would be good to then have training of other staff or supportive discussions on how to deal with said event...but i don’t think compulsory staff training to help the staff involved prior is necessarily needed. But perhaps there needs to be infrastructure there to deal with it if it happens

Training good in theory not sure where the time comes from

All

Support from within the team to colleagues engenders unison

I would agree if it was veterinary employer rather than veterinary practitioner

I think it will also be important to ensure professionals/experts on the topic are brought in to provide these training using, wherever possible, evidence-based approaches. I imagine role-playing might also be helpful so colleagues can familiarize themselves with terminology, phrasing, and practicing broaching conversations, etc..

Whilst I agree it would be very helpful for clients to be onboard pre-emptively regarding the risk of an adverse event affecting their pet, an adverse event is exactly that - a rare occurrence that maybe should not have happened, an error rather than an acknowledged complication or well recognised, known risk. Is it possible that discussing the risks of an adverse event occurring might undermine the clients' confidence in the veterinary practitioners' capabilities? I don't know the answer, it's just a thought.

needs to be inclusive of different personalities and neurodiversity, one approach may not work for all.

I think number 48 should fit within the normal communications teaching at university, along with taking a history and breaking bad news for example.

Need good communication to clients

I'm unclear whether 48 refers to communicating the risk of adverse events on the patient or the professional- I'm assuming it refers to risks to the patient

I do think 48 is important, but would expect that to be part of general client communication training

Communicating the risk of adverse events to clients is something that we should be more open about as practices. But I feel there is always great pressure to reassure owners that worst case scenario won't happen. As much as reassurance is important, discussing things that can be exceptions is important and we all tend to do it if adverse events are common but no so much when they are a bit rarer.

This training is widely provided in other medical professions.

I have disagreed with many of these as I cannot see a practical way of implementation. I have been trying to set aside 45 minutes for a meeting on a Friday and achieve it about 1:4 attempts. Meeting. Training. Meeting . Training. When does work happen. Excellent ideas impractical in practice.

"by veterinary practitioners" - as opposed to who? what's the other option? someone external?

I am uncertain about whether a display on adverse events in reception is of benefit. I have concerns it would lead to excessive queries/time demands for front desk staff who at or may not be equipped/trained to deal with it. On balance I feel better discussed within the consult room/on admission by clinical staff. Could also be covered off in consent forms.

Anonymity encourages the traumatised or anxious colleague

I read Veterinary Practioner Groups as a bit like AA meetings so for Q10 I scored 4 as you get more benefit from these types of meeting being there in person, reading meeting notes afterwards has minimal effect to help how people are feeling. However, if it was an in-practice meeting of all vets that work together, meeting minutes after important to know future practice policies etc so you can learn from adverse events

Hard to have 'should' options as no two events are similar and one set of rules may not fit everything.

buddy systems can work very well but they are specific to the personality types and the needs of each person can vary widely. they can also limit where a person can seek support if the expectation is that they rely on their buddy. I think they can be useful as part of a new team induction, if the relationship is mutually helpful and good it will naturally continue but i can envisage them not working simply due to personality types and differing needs of individuals- they work when they work but not sure they will in every circumstance. 16) anonymity is important in some cases but I think people often need to feel safe to speak up and they also potentially need to be part of the solution.

Adverse event review may also need to occur at random times in response to an adverse event and it should not be delayed until the following review.

Displaying policies is not very applicable to ambulatory equine practice. Something like this could be included on the reverse of invoices perhaps.

might be tricky if you are 'buddied' with someone who isn't engaging in these types of discussions, these need to be with the right person and this might be different for different people? For 19 and 18 I am not entirely sure what 'pre-agreed by veterinary practitioners within a practice' means, does this mean all vets need to agree on all of the particulars, or just that all vets need to be told what the roles/processes etc are before they are needed - the latter is more necessary, for large practices consensus may never be reached if everyone's opinion is factored into its creation, ideally I think someone trained in this area should design and create the specifics and then all the team should be able to provide anonymous feedback on how it is working.

rather than at the time they occur?

For corporate practices it may be advisable to discuss "adverse event charters" with the relevant teams involved (PSS support, Clinical Board, Regilatory advisers) - also - although pre-designating agrees roles and responsibilities would seem a sensible, logical thing to do I think it maybe could give

colleagues a fakers sense of security if they think the practice manager or lead vet will sort out any issues for them- these people aren’t always available and teams should ideally be trained and be prepared to engage in communication about adverse events with clients

In my work we have our team leaders as a kind of buddy and monthly 1:1 meeting where all aspects of life private and work are discussed. We also always have them as point of contact for any issues that we need to report. It means we have a safe place to talk about things by someone who understands and can help. I’d prefer this to anonymous messages as I think it’s hard to help someone if we don’t know who they are. There should be trust, a safe place and time to discuss important matters.

I'm not sure where the time will be found in the course of a busy working week to have these meetings

It's difficult to know how strongly I agree with statement 10 (in isolation) without knowing what is involved. Presumably content and delivery will have a huge effect on how useful these meetings would be. I could imagine that group discussions could potentially be harmful/have a negative effect if done poorly. Statement 17 regarding anonymity is a tricky one. Ideally you would want to create a culture where this is not necessary, in which clinicians feel able to talk honestly and openly with the rest of the team, but I appreciate this is not always the case. If the option of anonymity allows people to feedback information, they wouldn't otherwise then that is a positive thing, however I would be concerned that it might open the door for unkind or unfounded comments that might not be constructive and could potentially be quite harmful. I'm not quite sure of the difference between statements 18, 19 & 21. Statement 24 - I think it's important that every team member feels empowered to be able to have these conversations with clients, including the nursing staff. Whilst I appreciate some situations benefit from the involvement of a senior manager, I'm not sure this person necessarily needs to be the person who communicates to the client.

Not sure about a group meeting. I would not be opening up in front of a group.

I think this may depend on the individual and the circumstances. Sometimes a group meeting may be appropriate on other occasions an individual/ private meeting may be more appropriate

Buddy system and a clear well established culture within practices should hopefully mean this happens without the need for it to be specifically designated.

Normalising discussion reduces the stigma or fear of perfectionism gaining prominence

They should be spoken to first by the meeting facilitator so that they don't feel attacked in a larger group meeting

I can see this could be helpful for some people but it should be optional with no pressure applied for someone who does not wish to do this

one to one discussion may be more appropriate for some personalities, those with neurodiversity or mental health issues. The group aspect relies on psychological safety within the practice.

I am not sure if a group meeting would be the best approach for everyone

Although undoubtedly this helps it is hard to achieve in practice….I think

I believe the adverse events should be discussed and resolved privately in the first instance. Only once resolved should they be discussed in a group meeting.

Again, the format and delivery of this discussion will have a huge bearing on whether or not I feel it should happen. In answering this question (in isolation) in the way I have, I have assumed that the person leading the session has had suitable training and that the discussion is conducted in a kind way, without judgement or blame.

perhaps they should be led by a trained facilitator and someone involved with/high up in the practice ie: practice manager, clinical director. 36: perhaps they should be considered where relevant but not used to tar all other issues with the same brush, not used as "ammo" against the vet/nurse involved in the original adverse event?

I think many of these points have merit - but I think that how appropriate they are to individual cases may depend on the event, and the circumstances so I would like to a structure that can flex to the needs to the individual circumstances, otherwise there is a risk that all adverse events will be delat with in a standardised way, even if not appropriate in the individual circumstances

Vetsafe covers a lot of this off anyway. I am uncertain whether all discussions ought to be for all staff, and would suggest the wishes of the individual involved ought to be taken into account on a case-by-case basis before "public" meetings are the default position

Adverse events can't be prevented. Acknowledging that everyone will be on the receiving end at some stage helps teamwork and a supportive workplace

I don't think adverse events should be used in reviews etc otherwise people won't record them but if they have occurred due to negligence then surely they should be dealt with in a more formal way?

Still difficult to do this in a small practice setting

My hesitation regarding question 36 - I feel this depends on the context of the adverse event. If a colleague engages with the adverse event procedure and review and if human error was involved and the person/people involved engage and appear to learn from the incident, this seems the optimal outcome. However, if a person or the people involved do not engage in the process and do not acknowledge any possible human error and therefore do not seem to be willing or able to learn from the event, this would be concerning and, I feel, would warrant being addressed in performance review or disciplinary proceedings in the event of something that necessitated that level of intervention.

agree however where wilful, dishonest or illegal conduct is taking place adverse event details may form part of information which would apply for disciplinary matters e.g. deliberate harm caused to patients (can't turn a blind eye to serious misconduct matters or illegal activity)

For isolated adverse events I agree with this statement, however my answer reflects that if the same veterinary practitioner is repeatedly involved in adverse events, then this may have to be considered in a performance context for the safety of patients.

This requires psychological safety within the practice.

this might have exceptions e.g. individuals who are hindering discussion can be asked to leave and if a staff member is also an owner involved etc. Again 36 is a good rule of thumb to keep things blame-free and encourage participation and honesty, but there might be rare exceptions e.g. where severe misconduct has been identified, such as working drunk. For 49 I think something like VetSafe should be offered but not enforced, some corporates might have there own systems in place that work better for them and have more staff engagement, but ideally every veterinary professional should be able to learn from adverse events, not just those within the same practice/group (like the way aviation share their investigations and near misses to other airlines around the world, if the data is shared adequately e.g. VDS are open to sharing, it can improve safety more quickly).

I think engagement with centralised adverse event reporting systems should be a mandated part of clinical governance requirements!

it may be appropriate in some circumstances to discuss adverse events as part of a disciplinary, obviously only if there has been a deliberate error or somebody has not followed procedure or process. Q34, whilst ideal to have a trained facilitator, I would suggest they are not numerous and should not preclude having a meeting

Where adverse events are linked to conduct or poor performance it seems illogical for these not to be considered at performance reviews. If adverse events are discussed and dealt with separate to performance reviews then any improvement should form part of an individual performance review…I think

Reviewing meaning once the issue is resolved and learnings are being drawn

If same adverse event keeps hapenning to same vet then should be considered for performance management.

reporting adverse incidents can help reduce exposure in the future or can shape practice.

This has potential to adversely affect the rest of the team. Increase their workload. Generate resentment and lead to further adverse events

Perhaps this depends on the size of the adverse event and the impact it had. Question then follows if the salary should be adjusted if a vet/nurse can't carry out certain duties that they qualified for in the first place. Perhaps one way to approach this is in a stepwise/graduated manner over time.

I am not totally against this, but there is potentially an issue with requests for duties to be adjusted leading to excessive constraints on running the business, so in order to avoid this than anyone requesting this must be clear about the potential impact on colleagues' workloads/reduced service levels etc, and would suggest time limits are agreed in advance of changes being made.

Sensitivity is key. This event could be the last straw in someone who has a height stress challenge from a variety of other sources. Confidentiality needs to be considered

I agree with this where the key word is reasonable - it is very likely to be case by case specific

Where possible, depends on length of time, absolutely for short term but may be more difficult long term & put more stress in other colleagues

Though this is very important, it is also very hard to achieve in reality with current staff shortages

Do not believe it is helpful to avoid eg surgery after adverse surgical event - creates more fear of returning to the task and makes colleague feel you are judging them not to be competent for the task

This is important

Has to be for a fresh reasonable time frame and fair on the rest of the team

This has to be where possible as this cannot always be facilitated especially where an incident may affect multiple staff members.

reasonable adjustments are a specific term from Equality Act for disability adjustments - sometimes emotional impact may contribute to disability but not always

Very interesting questions and ideas to consider. In my experience of clinical practice then there is usually quite an obvious culture around adverse events which one can pick up on fairly quickly within teams.

Getting there in terms of refinement. Bring it on!

Thank you, very interesting. Good aspirations to have, just rolling it out to clinical practice may be challenging in the current climate of burnout, over-working, staff shortages.

I am finding this an interesting process answering questions as a practice owner when my personal experience of adverse event was as a young employee. It is helpful to reflect upon how I would Have felt as an employee vs how I now feel as an employer.

The process sounds good but the problem is making time to sit and discuss these things

Thank you- this is really interesting!!

Round 3

Very interesting to participate in. The measures discussed here ought to be tied in with thinking carefully about the selection of students for the degree, and also the universities' role in preparing students for general practice.

I feel it is important that a good level of optimism is present throughout the process. The expectation that things can and will go wrong but that we can cope with them. An ethos where we are all stretched and where achievements are celebrated.
